# Supplementary material for: Multimodal deep learning integration of cryo-EM and AlphaFold3 for high-accuracy protein structure determination
Source: Commun Chem. 2025 Oct 31;8:320. doi: 10.1038/s42004-025-01718-5 (PMC12579259; doi:10.1038/s42004-025-01718-5)
Supplement: Supplementary file 2 — Supplementary information [file 42004_2025_1718_MOESM2_ESM.pdf]

Multimodal deep learning integration of cryo-EM and AlphaFold3 for high-accuracy protein structure determination

Rajan Gyawali[1,2], Ashwin Dhakal[1,2], Jianlin Cheng[1,2,*]

[1]Department of Electrical Engineering and Computer Science, University of Missouri, Columbia, MO, 65211, United States

[2] NextGen Precision Health, University of Missouri, Columbia, MO, 65211, United States

*Corresponding author: Jianlin Cheng, E-mail: chengji@missouri.edu

This supplementary document consists of Supplementary Figures S1-S2 and Supplementary Tables S1-S8.

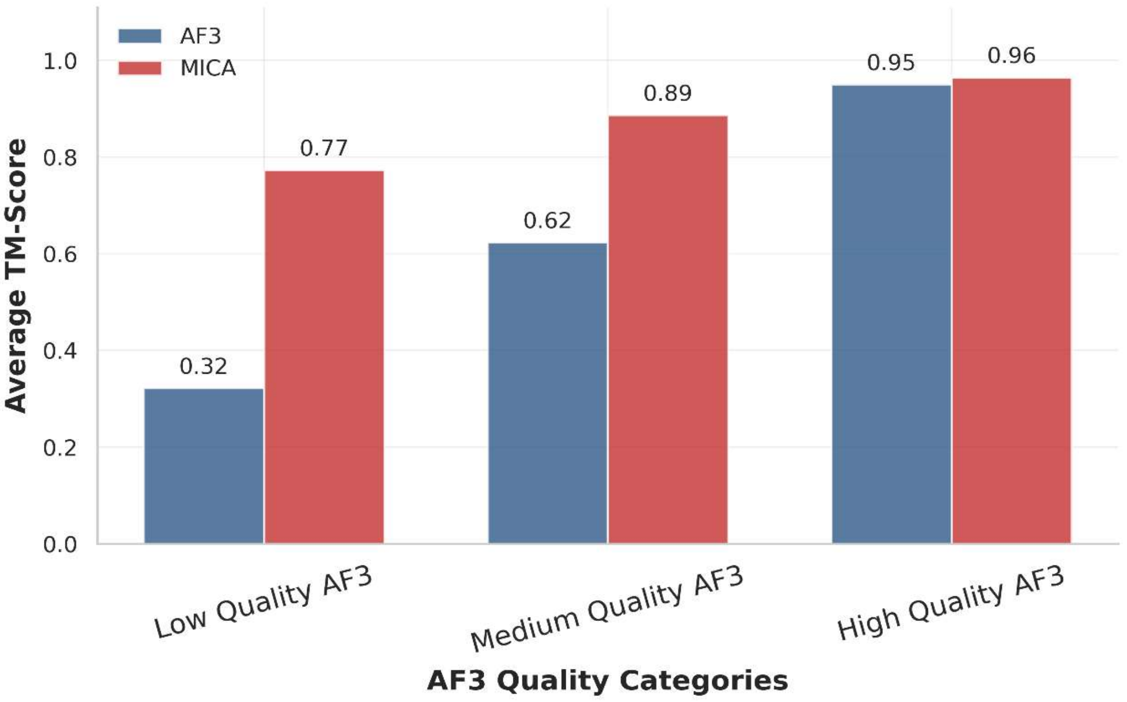

Supplementary Figure S1: Average TM-score comparison between MICA models and AlphaFold3 (AF3) predicted models over varying quality. The quality of AF3 models are categorized into three categories based on the TM-score as: low quality AF3 models (TM-score < 0.5, n=39 maps), medium quality AF3 models (0.5 ≤ TM-score < 0.8, n=38 maps), and high quality AF3 models (TM-score ≥ 0.8, n=83 maps).

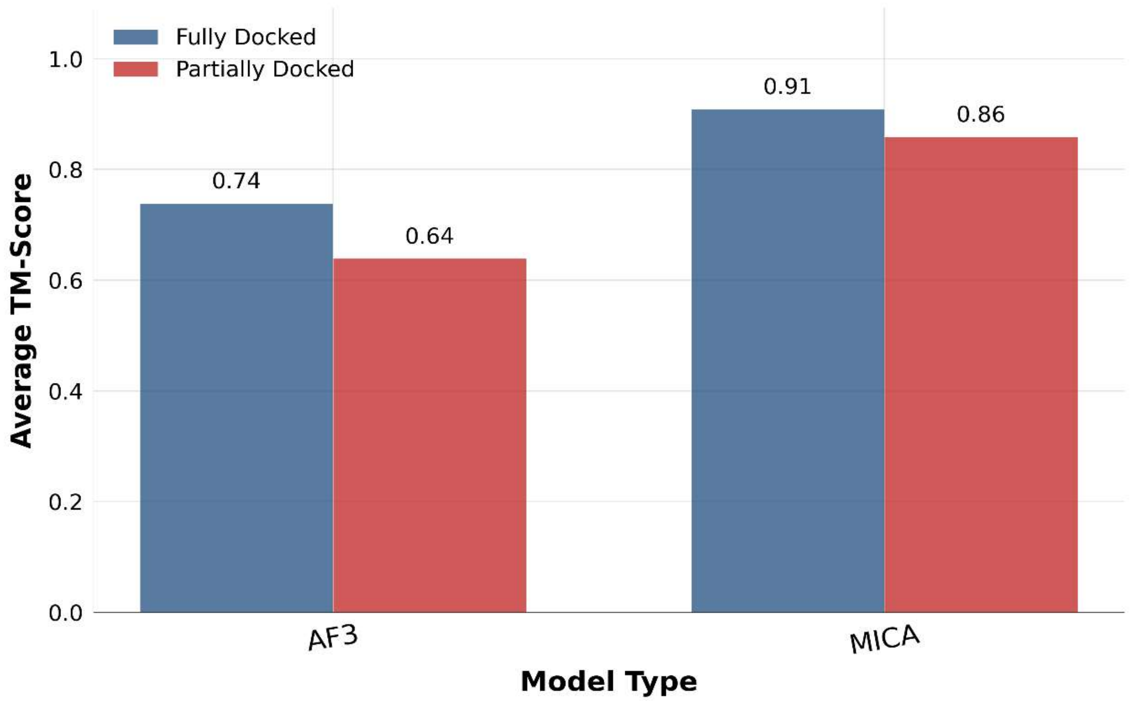

Supplementary Figure S2: Performance comparison of MICA to assess the impact of docking quality. The blue boxes represent the average TM-score for fully docked AlphaFold3 models (domains) into cryo-density maps and red boxes represent partially docked AlphaFold3 models into cryo-EM density maps.

Supplementary Table S1: Performance metrics of MICA against ModelAngelo and EModelX(+AF) on Cryo2StructData test dataset

EMD ID	Model Type	TM-Score	Cα Match	Cα Quality Score	Mean Length	Predicted Length	Reference Length	Aligned Cα Length	Sequence Identity	Sequence Match
15691	EModelX(+AF)	0.982	97.9	100.354	48.8	818	798	788	1	98.5
15691	MICA	0.973	98.1	100.559	52.2	818	798	784	0.986	96.9
15691	ModelAngelo	0.842	93.6	88.439	62.2	754	798	674	0.985	98.4
16110	EModelX(+AF)	0.997	100	100.663	117.3	1063	1056	1056	0.727	72.4
16110	MICA	0.998	99.7	100.739	351	1067	1056	1056	0.73	72.4
16110	ModelAngelo	0.944	95.2	89.971	41.9	998	1056	998	0.976	97.9
16112	EModelX(+AF)	0.998	99.8	100.643	265.8	1074	1065	1065	1	99.7
16112	MICA	0.997	100	100.939	355	1075	1065	1063	1	100
16112	ModelAngelo	0.989	99.2	98.175	176	1054	1065	1054	0.995	99.1
16140	EModelX(+AF)	0.98	96.4	97.362	103.1	1620	1604	1589	0.974	98.1
16140	MICA	0.986	98.4	98.584	112.7	1607	1604	1597	0.981	97.3
16140	ModelAngelo	0.89	93.4	86.878	62.4	1492	1604	1430	0.991	98.5
16328	EModelX(+AF)	0.996	100	118.692	321	1143	963	962	1	100
16328	MICA	0.998	99.7	118.646	192	1146	963	963	1	99.7
16328	ModelAngelo	0.993	99.4	105.283	159.5	1020	963	957	0.997	99.7
16332	EModelX(+AF)	0.96	97.6	114.337	63	1134	968	943	0.964	96.2
16332	MICA	0.985	99.1	113.637	137	1110	968	958	0.994	99.2
16332	ModelAngelo	0.859	95	91.271	76.5	930	968	836	0.99	99.7
16333	EModelX(+AF)	0.997	100	114.827	308	1061	924	923	1	100
16333	MICA	0.998	100	109.632	308	1013	924	924	1	100
16333	ModelAngelo	0.994	99.5	101.008	153.2	938	924	919	0.998	99.8
16475	EModelX(+AF)	0.378	52.5	28.076	12.2	1353	2530	1011	0.795	62.5
16475	MICA	0.702	76.2	59.002	20.3	1959	2530	1820	0.902	84.6
16475	ModelAngelo	0.95	95.3	91.044	86.1	2417	2530	2406	0.991	98.9
17360	EModelX(+AF)	0.809	82.8	68.307	178.7	2135	2588	2118	0.961	99.1
17360	MICA	0.843	99.8	100.301	64.6	2601	2588	2244	0.953	97.7
17360	ModelAngelo	0.449	85.6	64.101	32.6	1938	2588	1164	0.971	98.2
17402	EModelX(+AF)	0.989	97.7	102.737	53.5	2305	2192	2181	0.992	95.6
17402	MICA	0.994	99.1	104.661	55.7	2315	2192	2188	0.989	97.5
17402	ModelAngelo	0.748	86.5	75.293	40.4	1908	2192	1693	0.949	97.2
17429	EModelX(+AF)	0.997	100	100	526	1052	1052	1052	1	99.6
17429	MICA	0.994	99	99	148.9	1052	1052	1052	1	99.1
17429	ModelAngelo	0.989	99.6	99.789	174.7	1054	1052	1041	0.996	99.4
17574	EModelX(+AF)	0.98	98.2	97.79	117.6	954	958	944	0.989	98.6
17574	MICA	0.995	99.4	101.579	105.8	979	958	956	0.988	98.1
17574	ModelAngelo	0.94	94.5	139.777	50.3	1417	958	902	0.981	97.8
17575	EModelX(+AF)	0.961	97.5	97.704	51.8	958	956	928	0.984	95.9
17575	MICA	0.831	83.6	79.84	88.8	913	956	798	0.987	98.2
17575	ModelAngelo	0.445	75.9	85.824	16.5	1081	956	429	0.942	93.9
17958	EModelX(+AF)	0.243	48	23.247	7.7	895	1848	491	0.802	44.5
17958	MICA	0.313	67.2	45.455	13.8	1250	1848	616	0.948	72.4
17958	ModelAngelo	0.081	95.5	91.211	73.5	1765	1848	149	0.993	99.3
17961	EModelX(+AF)	0.115	12.6	1.596	17.3	523	4128	490	0.882	81
17961	MICA	0.253	51.6	26.675	19.6	2134	4128	1138	0.776	80.7
17961	ModelAngelo	0.042	99.1	98.5	170.4	4103	4128	172	0.994	100
17964	EModelX(+AF)	0.249	24.9	6.262	168	339	1348	336	0.982	97.9
17964	MICA	0.736	73.7	54.619	198.6	999	1348	994	0.991	98.8
17964	ModelAngelo	0.25	99.8	99.578	336.2	1345	1348	337	1	100
17966	EModelX(+AF)	0.503	59.2	35.184	33.3	1172	1972	1008	0.964	89.5
17966	MICA	0.732	80.3	64.501	52.8	1584	1972	1445	1	91.7
17966	ModelAngelo	0.246	98.2	96.457	215.2	1937	1972	485	0.994	99.7
18148	EModelX(+AF)	0.988	98.9	98.148	146.4	1175	1184	1175	0.997	99.1

18148	MICA	0.994	99.7	99.447	196.7	1181	1184	1180	0.993	99.7
18148	ModelAngelo	0.989	99.2	98.278	117.4	1173	1184	1173	0.998	99.5
18212	EModelX(+AF)	0.993	99	98.555	125.9	2658	2670	2658	1	99.8
18212	MICA	0.996	99.3	99.077	165.6	2664	2670	2664	0.999	99.6
18212	ModelAngelo	0.986	98.7	99.772	62.7	2699	2670	2636	0.989	98.6
18298	EModelX(+AF)	0.975	99.6	98.522	230	457	462	456	0.989	100
18298	MICA	0.992	99.6	99.169	230	460	462	460	1	100
18298	ModelAngelo	0.585	81.4	64.662	20.9	367	462	271	0.974	97.3
18300	EModelX(+AF)	0.604	78.4	53.891	11.4	763	1110	715	0.761	64.4
18300	MICA	0.985	98.6	101.087	64.4	1138	1110	1102	0.972	96.2
18300	ModelAngelo	0.478	96.8	93.835	89.5	1076	1110	532	0.998	99.6
18415	EModelX(+AF)	0.982	98.8	103.499	101.2	859	820	813	0.982	97.8
18415	MICA	0.99	99.5	101.199	204	834	820	814	0.998	99.8
18415	ModelAngelo	0.956	97.3	93.384	66.5	787	820	785	0.99	99.5
18436	EModelX(+AF)	0.996	99.2	98.985	107.8	1844	1848	1844	0.998	99
18436	MICA	0.996	99.2	98.985	141	1844	1848	1844	0.998	99.3
18436	ModelAngelo	0.975	97.5	95.284	75.1	1806	1848	1804	0.988	98.8
18634	EModelX(+AF)	0.946	92.7	92.346	28.5	1042	1046	1015	0.939	91.3
18634	MICA	0.938	92.4	90.722	30.2	1027	1046	1008	0.932	91.1
18634	ModelAngelo	0.425	77.1	59.483	33.6	807	1046	447	0.987	97.3
18635	EModelX(+AF)	0.872	88.9	85.33	15.5	1004	1046	948	0.885	81.5
18635	MICA	0.943	93.5	93.947	28.8	1051	1046	1010	0.904	89.8
18635	ModelAngelo	0.415	81.1	64.585	26.5	833	1046	437	0.97	96.2
26165	EModelX(+AF)	0.997	99.8	99.8	514	1030	1030	1030	1	100
26165	MICA	0.998	99.8	99.8	514	1030	1030	1030	1	100
26165	ModelAngelo	0.959	95.9	92.269	61.8	991	1030	990	0.994	99.2
28628	EModelX(+AF)	0.934	95.5	96.052	52.6	1219	1212	1145	0.985	96.3
28628	MICA	0.967	96.1	105.536	83.2	1331	1212	1183	0.997	99.6
28628	ModelAngelo	0.878	96.8	94.404	90.2	1182	1212	1066	0.999	99.9
28994	EModelX(+AF)	0.98	95.8	95.907	35.9	2698	2695	2665	0.99	95.5
28994	MICA	0.953	94.2	91.019	43.8	2604	2695	2584	0.991	95.8
28994	ModelAngelo	0.854	86.1	74.758	33.6	2340	2695	2306	0.977	95.9
29069	EModelX(+AF)	0.891	86.7	83.794	22.1	1874	1939	1761	0.961	88.2
29069	MICA	0.939	91.7	91.558	26.6	1936	1939	1858	0.97	90.9
29069	ModelAngelo	0.368	65.9	44.149	20.9	1299	1939	717	0.943	93.8
29551	EModelX(+AF)	0.68	73.9	58.238	20.2	885	1123	794	0.897	83.3
29551	MICA	0.901	89.5	90.616	20.5	1137	1123	1051	0.888	87.2
29551	ModelAngelo	0.533	71.1	51.6	23.5	815	1123	600	0.978	98
29552	EModelX(+AF)	0.889	91.6	86.043	25.2	929	989	898	0.93	89.6
29552	MICA	0.936	95.2	95.874	33.6	996	989	937	0.962	94.5
29552	ModelAngelo	0.512	80	65.197	17.2	806	989	509	0.963	95.4
29823	EModelX(+AF)	0.709	75.3	62.37	30.5	1341	1619	1196	0.954	90.5
29823	MICA	0.971	97.5	102.498	35.1	1702	1619	1592	0.96	94.9
29823	ModelAngelo	0.629	78.8	63.809	27.7	1311	1619	1042	0.955	95.8
29930	EModelX(+AF)	0.993	99.2	98.779	100.1	1408	1414	1408	0.999	98.9
29930	MICA	0.994	99.3	98.949	234	1409	1414	1409	0.999	99.9
29930	ModelAngelo	0.878	98.2	97.436	63.1	1403	1414	1243	0.991	99.1
33592	EModelX(+AF)	0.799	86.2	76.493	20.5	1647	1856	1525	0.915	86.9
33592	MICA	0.956	96	94.707	55.7	1831	1856	1789	0.985	97
33592	ModelAngelo	0.905	95.6	90.964	63.4	1766	1856	1684	0.997	98.8
33639	EModelX(+AF)	0.992	99.3	98.563	156	3744	3772	3744	1	100
33639	MICA	0.992	99.3	98.563	156	3744	3772	3744	1	100
33639	ModelAngelo	0.992	99.3	98.589	156	3745	3772	3745	0.999	99.9
34219	EModelX(+AF)	0.957	94.9	92.854	22	1044	1067	1041	0.992	95.8
34219	MICA	0.988	98.7	98.515	105.3	1065	1067	1065	0.999	99.5
34219	ModelAngelo	0.627	77.8	61.978	14.6	850	1067	676	0.953	94.3

34275	EModelX(+AF)	0.961	97.4	96.127	53.1	755	765	739	0.955	95.2
34275	MICA	0.917	91.9	100.79	70.2	839	765	710	0.986	98.6
34275	ModelAngelo	0.987	98.6	98.729	94.2	766	765	756	0.999	100
34304	EModelX(+AF)	0.986	97.8	97.697	28	944	945	944	0.999	95.1
34304	MICA	0.983	97.2	96.994	34	943	945	942	0.995	94.6
34304	ModelAngelo	0.719	82.9	67.197	26.1	766	945	683	0.988	96.9
34368	EModelX(+AF)	0.992	99.4	98.85	251.6	1258	1265	1258	0.998	99.6
34368	MICA	0.994	99.5	99.107	314.8	1260	1265	1260	0.999	99.8
34368	ModelAngelo	0.992	99.1	98.552	139.3	1258	1265	1256	0.996	99.6
34369	EModelX(+AF)	0.993	99.4	99.025	219.7	1321	1326	1321	1	99.4
34369	MICA	0.992	99.3	98.776	219.5	1319	1326	1318	0.995	99.3
34369	ModelAngelo	0.987	98.7	97.583	119	1311	1326	1311	0.997	99.2
34412	EModelX(+AF)	0.72	85.6	73.786	18.3	1474	1710	1300	0.821	80.3
34412	MICA	0.918	91.2	89.44	32.5	1677	1710	1599	0.951	95.4
34412	ModelAngelo	0.811	83.4	69.939	32.4	1434	1710	1390	0.978	96.2
34504	EModelX(+AF)	0.997	99.9	99.748	984	1968	1971	1968	1	100
34504	MICA	0.999	99.9	99.849	492	1970	1971	1970	1	100
34504	ModelAngelo	0.994	99.5	98.894	245	1959	1971	1959	0.998	99.8
34825	EModelX(+AF)	0.994	98.8	100.632	71	1318	1294	1292	0.977	96.9
34825	MICA	0.981	98.1	100.071	48.8	1320	1294	1281	0.993	95.7
34825	ModelAngelo	0.518	96	92.958	56.5	1253	1294	672	0.987	97.6
35075	EModelX(+AF)	0.995	99.3	110.621	101.7	2052	1842	1840	0.974	97.7
35075	MICA	0.994	99.3	105.607	101.7	1959	1842	1838	0.975	98
35075	ModelAngelo	0.925	95.1	91.228	48.7	1767	1842	1709	0.987	97.9
35452	EModelX(+AF)	0.982	99	97.449	131.7	3142	3192	3142	0.991	99.5
35452	MICA	0.983	98	96.649	136	3148	3192	3148	0.987	99
35452	ModelAngelo	0.915	99.5	99.344	132.3	3187	3192	2925	0.987	99.5
35453	EModelX(+AF)	0.985	99.7	101.351	127.6	3253	3200	3158	0.993	98.4
35453	MICA	0.997	99.8	101.671	199.5	3260	3200	3192	0.997	99.7
35453	ModelAngelo	0.943	99.1	99.719	113.3	3220	3200	3019	0.994	99.5
35454	EModelX(+AF)	0.979	98.5	99.547	73.3	3234	3200	3141	0.992	98
35454	MICA	0.997	99.6	101.592	159.4	3264	3200	3192	0.997	99.7
35454	ModelAngelo	0.853	96.8	94.229	77.4	3115	3200	2733	0.988	98.2
35621	EModelX(+AF)	0.953	94.7	90.687	54.6	3367	3516	3367	0.982	96.8
35621	MICA	0.976	96.4	94.563	46.4	3449	3516	3449	0.976	95.2
35621	ModelAngelo	0.902	91.9	83.144	80.8	3181	3516	3174	0.99	98.8
35713	EModelX(+AF)	0.317	31.8	10.232	61.8	250	777	248	0.956	95.1
35713	MICA	0.297	31.4	10.063	34.9	249	777	233	1	93.9
35713	ModelAngelo	0.323	95.5	91.198	92.8	742	777	251	0.996	99.2
36060	EModelX(+AF)	0.986	99	97.824	79.2	3162	3200	3162	0.99	97.2
36060	MICA	0.99	99	98.258	132	3176	3200	3176	0.987	99
36060	ModelAngelo	0.986	99	99.062	99	3202	3200	3160	0.986	98.5
36150	EModelX(+AF)	0.901	91.7	84.581	65.5	2899	3143	2875	0.975	96.8
36150	MICA	0.91	91.9	85.584	67.2	2927	3143	2892	0.973	97.8
36150	ModelAngelo	0.987	98.7	97.444	110.8	3103	3143	3103	0.994	99.5
36391	EModelX(+AF)	0.942	94.4	91.869	50.3	726	746	707	0.959	96
36391	MICA	0.93	94.1	111.381	58.5	883	746	701	0.959	97.4
36391	ModelAngelo	0.993	99.3	102.362	82.3	769	746	741	0.997	99.7
36628	EModelX(+AF)	0.981	98.7	97.931	126.7	382	385	380	0.992	99.2
36628	MICA	0.985	98.7	97.931	190	382	385	382	1	99.5
36628	ModelAngelo	0.892	96.9	93.88	124.3	373	385	344	1	99.7
36637	EModelX(+AF)	0.984	98.7	103.07	63.2	401	384	383	1	97.6
36637	MICA	0.979	97.7	99.99	75	393	384	380	0.997	98.7
36637	ModelAngelo	0.896	95.6	91.617	122.3	368	384	345	1	99.5
36661	EModelX(+AF)	0.907	92.3	90.152	41.1	1217	1246	1158	0.957	93.9
36661	MICA	0.925	95.2	99.555	29.6	1303	1246	1200	0.906	90.2

36661	ModelAngelo	0.451	84.1	72.018	65.5	1067	1246	565	0.979	98.5
36662	EModelX(+AF)	0.965	97.1	96.312	54.8	1345	1356	1320	0.987	96.5
36662	MICA	0.954	95.2	95.06	49.7	1354	1356	1306	0.989	96.4
36662	ModelAngelo	0.533	94.2	90.379	60.8	1301	1356	724	0.981	97.3
36856	EModelX(+AF)	0.968	97.9	103.174	67.5	1526	1448	1409	0.996	97.5
36856	MICA	0.957	97.7	101.681	64.3	1507	1448	1395	0.999	97.2
36856	ModelAngelo	0.946	97	97.938	63.8	1462	1448	1376	0.988	99.6
36863	EModelX(+AF)	0.918	93.9	93.156	43.5	1377	1388	1283	0.991	95.8
36863	MICA	0.925	95.5	95.913	36.8	1394	1388	1293	0.987	94
36863	ModelAngelo	0.747	90.5	81.698	36.9	1253	1388	1045	0.969	98.2
36864	EModelX(+AF)	0.921	94.4	95.898	40.9	1408	1386	1284	0.981	95.4
36864	MICA	0.92	92.4	90.667	49.3	1360	1386	1284	0.982	97
36864	ModelAngelo	0.754	88	78.222	40.7	1232	1386	1049	0.979	99
37235	EModelX(+AF)	0.991	98.7	98.295	64.1	2909	2921	2908	0.997	96.6
37235	MICA	0.991	98.5	98.028	68.5	2907	2921	2906	0.998	97
37235	ModelAngelo	0.991	99	99.576	60.2	2938	2921	2901	0.973	96.6
40039	EModelX(+AF)	0.978	98.5	98.35	72	657	658	648	0.972	96.5
40039	MICA	0.992	98.8	99.551	130	663	658	658	1	99.1
40039	ModelAngelo	0.953	95.1	90.909	44.7	629	658	629	0.981	98.4
40040	EModelX(+AF)	0.988	99.5	100.26	72.4	1320	1310	1297	0.982	96.9
40040	MICA	0.985	99.7	100.613	108.8	1322	1310	1294	0.99	97.9
40040	ModelAngelo	0.946	97.7	95.612	75.3	1282	1310	1240	0.994	99.1
40041	EModelX(+AF)	0.999	100	100.302	662	3984	3972	3972	1	99.8
40041	MICA	0.994	99.8	99.825	165.2	3973	3972	3954	0.999	99.2
40041	ModelAngelo	0.995	99.5	99.024	232.5	3953	3972	3953	0.998	99.8
40063	EModelX(+AF)	0.32	35.1	12.675	16.9	260	720	236	0.843	77.9
40063	MICA	0.265	33.2	11.251	26.6	244	720	191	1	92.5
40063	ModelAngelo	0.319	92.8	86.871	60.7	674	720	231	0.974	99
40229	EModelX(+AF)	0.964	94.1	92.194	54.2	1693	1728	1693	0.998	98.2
40229	MICA	0.974	95.9	94.457	66.3	1702	1728	1702	0.994	99.5
40229	ModelAngelo	0.938	96.8	94.335	52.2	1684	1728	1626	0.988	100
40334	EModelX(+AF)	0.977	99.1	108.432	44.2	488	446	442	0.984	95.7
40334	MICA	0.963	98.2	104.585	39.8	475	446	439	0.986	94.1
40334	ModelAngelo	0.477	84.3	72.392	26.9	383	446	216	0.991	95.5
40335	EModelX(+AF)	0.944	92.5	96.584	26.2	473	453	443	0.975	93.6
40335	MICA	0.982	97.1	100.53	29.3	469	453	453	0.976	94.5
40335	ModelAngelo	0.347	62	39.007	12.8	285	453	159	0.962	95.7
40336	EModelX(+AF)	0.948	94.2	106.266	22.1	502	445	438	0.943	90.2
40336	MICA	0.982	99.1	110.458	73.5	496	445	444	0.993	98
40336	ModelAngelo	0.339	57.1	32.72	13.4	255	445	152	0.947	95.7
40339	EModelX(+AF)	0.986	98.7	105.987	44.1	480	447	446	0.98	97.5
40339	MICA	0.982	98.4	104.344	62.9	474	447	445	0.993	96.8
40339	ModelAngelo	0.49	85.2	73.192	31.8	384	447	221	0.986	99.2
40352	EModelX(+AF)	0.996	100	102	125	510	500	500	0.998	99.8
40352	MICA	0.992	99.6	102.588	99.6	515	500	500	0.992	98.6
40352	ModelAngelo	0.993	99.2	99.398	124	501	500	499	0.99	99.6
40354	EModelX(+AF)	0.948	95.1	95.1	31.3	494	494	482	0.929	92.8
40354	MICA	0.993	100	103.441	70.6	511	494	494	0.974	96.6
40354	ModelAngelo	0.446	78.5	61.815	25.9	389	494	221	0.959	96.1
40355	EModelX(+AF)	0.945	94.6	91.762	43	485	500	479	0.983	96.2
40355	MICA	0.988	98.8	100.776	61.8	510	500	500	0.978	97.4
40355	ModelAngelo	0.498	85.8	73.616	28.6	429	500	250	0.968	97.7
40889	EModelX(+AF)	0.964	96.9	98.678	64.6	1221	1199	1164	0.992	98.2
40889	MICA	0.952	94.8	102.153	75.8	1292	1199	1152	0.989	99
40889	ModelAngelo	0.976	97.7	96.152	90.1	1180	1199	1171	0.997	99.7
41066	EModelX(+AF)	0.94	98.7	101.257	42.3	396	386	366	0.962	92.4

41066	MICA	0.932	98.2	97.437	42.1	383	386	366	0.989	94.5
41066	ModelAngelo	0.878	95.6	91.637	41	370	386	340	0.994	99.5
41266	EModelX(+AF)	0.996	99.7	99.7	153.2	1230	1230	1230	1	99.7
41266	MICA	0.996	99.5	99.5	153	1230	1230	1230	1	99.2
41266	ModelAngelo	0.98	99	97.873	87	1216	1230	1208	0.991	99.3
41604	EModelX(+AF)	0.94	94.7	90.846	86.9	1320	1376	1299	0.995	98.2
41604	MICA	0.947	94.9	91.314	81.6	1324	1376	1308	0.995	98.3
41604	ModelAngelo	0.861	95.7	92.57	54.9	1331	1376	1186	0.991	99
41624	EModelX(+AF)	0.833	89.6	80.273	31.6	1420	1585	1336	0.974	90.5
41624	MICA	0.836	90	82.278	29.7	1449	1585	1339	0.993	91.3
41624	ModelAngelo	0.921	92.4	85.23	73.2	1462	1585	1462	0.994	100
41628	EModelX(+AF)	0.811	88.4	78.615	21.6	1414	1590	1312	0.963	89.7
41628	MICA	0.776	83	71.203	29.3	1364	1590	1257	0.962	92
41628	ModelAngelo	0.894	89.9	80.458	40.9	1423	1590	1423	0.993	99.3
41768	EModelX(+AF)	0.938	90.6	95.319	16.7	505	480	471	0.964	89.7
41768	MICA	0.968	96.2	101.411	33	506	480	476	0.987	95.5
41768	ModelAngelo	0.154	36.2	13.952	15.8	185	480	75	0.973	90.8
41946	EModelX(+AF)	0.996	99.8	99.494	223.5	3573	3584	3573	0.996	99.6
41946	MICA	0.997	99.8	99.577	223.5	3576	3584	3576	0.996	99.6
41946	ModelAngelo	0.998	100	100.223	448	3592	3584	3577	0.996	99.8

Supplementary Table S2: Performance metrics of MICA against ModelAngelo and EModelX(+AF) on standard test dataset

EMD ID	Model Type	TM-Score	Cα Match	Cα Quality Score	Mean Length	Predicted Length	Reference Length	Aligned Cα Length	Sequence Identity	Sequence Match
14066	EModelX(+AF)	0.85	87.5	83.996	14.2	839	874	777	0.927	84.2
14066	MICA	0.836	86.6	87.393	12.2	882	874	770	0.866	79
14066	ModelAngelo	0.166	55.1	31.017	12.1	492	874	146	0.959	92.7
14716	EModelX(+AF)	0.969	97.4	109.009	21	554	495	493	0.966	92.1
14716	MICA	0.976	97.4	105.271	25.4	535	495	495	0.935	91.5
14716	ModelAngelo	0.342	74.9	57.499	18.6	380	495	171	0.977	96.8
14725	EModelX(+AF)	0.937	92.5	102.176	17	528	478	468	0.942	89.8
14725	MICA	0.974	97.1	104.616	27.3	515	478	477	0.958	94.2
14725	ModelAngelo	0.26	55.6	31.522	11.6	271	478	126	0.952	91.7
14842	EModelX(+AF)	0.885	84.9	84.132	17	1862	1879	1713	0.907	85.9
14842	MICA	0.86	84.6	81.628	14.3	1813	1879	1685	0.872	80.4
14842	ModelAngelo	0.697	72.4	54.136	18.1	1405	1879	1320	0.934	91.6
14847	EModelX(+AF)	0.764	77.8	65.786	22.5	1599	1891	1509	0.874	85.8
14847	MICA	0.755	80.9	72.258	19.7	1689	1891	1485	0.875	83.2
14847	ModelAngelo	0.245	68.1	45.808	21	1272	1891	466	0.953	93.7
14848	EModelX(+AF)	0.837	84.7	77.501	22.7	2336	2553	2187	0.92	88.3
14848	MICA	0.834	85.8	79.213	25.4	2357	2553	2183	0.918	88.6
14848	ModelAngelo	0.425	75.4	56.646	39.5	1918	2553	1089	0.988	97.6
14869	EModelX(+AF)	0.793	78.2	63.072	44.5	8960	11109	8881	0.967	95
14869	MICA	0.796	87.7	77.153	48.5	9773	11109	8932	0.971	96.2
14869	ModelAngelo	0.623	75.5	50.993	54.8	7503	11109	6928	0.975	98.7
15378	EModelX(+AF)	0.934	95.7	93.39	76	2790	2859	2717	0.972	98.4
15378	MICA	0.997	99.8	101.685	285.3	2913	2859	2854	1	99.8
15378	ModelAngelo	0.995	99.5	102.041	284.5	2932	2859	2845	0.999	99.9
15540	EModelX(+AF)	0.402	86.7	51.685	11.9	1358	2278	967	0.923	85.4
15540	MICA	0.675	91.5	72.059	13.8	1794	2278	1579	0.946	92.4
15540	ModelAngelo	0.161	61.1	22.933	10.7	855	2278	369	0.946	78.2
15635	EModelX(+AF)	0.95	95.4	97.634	24.3	1617	1580	1522	0.985	91.4
15635	MICA	0.939	95.7	97.82	25.2	1615	1580	1505	0.973	91.1
15635	ModelAngelo	0.657	85.1	73.358	28.6	1362	1580	1045	0.979	97.9

15673	EModelX(+AF)	0.765	89.7	80.717	29.9	629	699	558	0.912	87.1
15673	MICA	0.747	88.4	74.489	29.4	589	699	546	0.93	88.8
15673	ModelAngelo	0.616	89.7	101.506	20.9	791	699	433	0.977	96.2
15684	EModelX(+AF)	0.914	94	93.162	29.7	1001	1010	950	0.947	90.5
15684	MICA	0.889	94.5	92.816	43.5	992	1010	915	0.972	93.7
15684	ModelAngelo	0.665	88.5	78.511	44.6	896	1010	674	0.991	98
15685	EModelX(+AF)	0.642	79.8	73.656	12.1	1067	1156	787	0.836	70.7
15685	MICA	0.819	83.3	77.103	28.3	1070	1156	963	0.945	91.9
15685	ModelAngelo	0.46	71.6	51.284	27.5	828	1156	535	0.987	98.8
15686	EModelX(+AF)	0.747	79.1	86.919	10.1	1056	961	814	0.797	68.6
15686	MICA	0.814	83.4	99.368	15.1	1145	961	865	0.875	74.5
15686	ModelAngelo	0.048	15.8	2.318	9.5	141	961	51	0.118	89.5
15785	EModelX(+AF)	0.49	58.8	46.877	9.8	1667	2091	1107	0.812	63.4
15785	MICA	0.478	64.9	55.309	9.2	1782	2091	1065	0.869	71.5
15785	ModelAngelo	0.318	39.9	14.96	18.4	784	2091	670	0.976	95.3
23544	EModelX(+AF)	0.997	99.5	100.202	246.7	2997	2976	2973	0.994	99.1
23544	MICA	0.992	99.1	99.5	122.8	2988	2976	2960	0.987	98.8
23544	ModelAngelo	0.972	98.9	98.202	184	2955	2976	2898	0.997	99.2
26595	EModelX(+AF)	0.721	99.5	90.545	132.7	2184	2400	1740	0.99	99
26595	MICA	0.947	99	99.701	79.2	2417	2400	2280	0.99	98.7
26595	ModelAngelo	0.63	98.8	189.943	131.7	4614	2400	1514	0.999	99
26754	EModelX(+AF)	0.93	93	92.354	25.9	2431	2448	2294	0.984	94.6
26754	MICA	0.923	90.1	89.033	30.2	2419	2448	2283	0.971	94.7
26754	ModelAngelo	0.3	76.6	59.046	23.4	1887	2448	738	0.969	97.1
26770	EModelX(+AF)	0.942	89.3	97.505	18.5	2448	2242	2161	0.97	89.3
26770	MICA	0.905	88.4	94.669	19.4	2401	2242	2085	0.925	89.6
26770	ModelAngelo	0.353	66.9	47.236	27.8	1583	2242	799	0.949	97.1
26782	EModelX(+AF)	0.632	99.7	71.944	198	451	625	397	0.995	99.5
26782	MICA	0.632	99.2	74.598	197	470	625	397	0.992	100
26782	ModelAngelo	0.311	98	61.309	64.8	391	625	195	1	99
26841	EModelX(+AF)	0.848	86.9	85.354	28.2	4748	4834	4146	0.954	91.5
26841	MICA	0.88	89.7	86.917	43.2	4684	4834	4285	0.968	94.9
26841	ModelAngelo	0.727	78.9	77.268	31.1	4734	4834	3542	0.96	97.4
26858	EModelX(+AF)	0.977	96.9	100.018	23.7	1187	1150	1140	0.927	88.3
26858	MICA	0.967	97.4	104.43	18.7	1233	1150	1128	0.92	87.5
26858	ModelAngelo	0.944	93.9	90.716	18	1111	1150	1100	0.95	89.8
26917	EModelX(+AF)	0.985	99.2	100.255	108.8	665	658	651	0.997	99.1
26917	MICA	0.997	99.4	102.572	130.8	679	658	658	0.995	99.5
26917	ModelAngelo	0.59	91.8	88.87	75.5	637	658	389	0.997	99.8
26948	EModelX(+AF)	0.984	98.6	99.05	162.2	661	658	649	1	99.8
26948	MICA	0.991	98.9	102.056	130.2	679	658	655	0.997	99.5
26948	ModelAngelo	0.662	98.9	101.305	162.8	674	658	436	1	100
26973	EModelX(+AF)	0.979	97.9	98.049	107.2	658	657	648	0.991	99.2
26973	MICA	0.995	99.7	102.432	93.6	675	657	657	0.994	99.2
26973	ModelAngelo	0.662	99.1	102.418	130.2	679	657	435	1	99.8
26974	EModelX(+AF)	0.989	98.9	100.95	114.5	1182	1158	1148	0.999	99.9
26974	MICA	0.909	98.1	99.625	113.6	1176	1158	1078	0.59	97.2
26974	ModelAngelo	0.921	95.9	92.919	74	1122	1158	1069	0.993	99.9
26976	EModelX(+AF)	0.58	87.7	56.344	25.5	469	730	441	0.896	89.8
26976	MICA	0.673	99.8	67.809	164.3	496	730	494	1	99.2
26976	ModelAngelo	0.606	89.1	54.192	27.5	444	730	444	0.993	99.3
26978	EModelX(+AF)	0.973	97.7	98.494	73.9	992	984	965	0.987	98.5
26978	MICA	0.989	98.7	101.91	64.7	1016	984	982	0.982	98.5
26978	ModelAngelo	0.957	95.9	95.997	78.7	985	984	943	0.993	99.6
26993	EModelX(+AF)	0.891	91	96.566	13.2	399	376	355	0.935	84.8
26993	MICA	0.883	91.5	91.987	24.6	378	376	352	0.98	92.4

26993	ModelAngelo	0.611	61.2	38.25	7.7	235	376	234	0.915	90.9
26994	EModelX(+AF)	0.617	87	64.77	12.9	472	634	400	0.99	90.3
26994	MICA	0.625	92.3	66.532	18.5	457	634	400	0.993	93.8
26994	ModelAngelo	0.287	88	49.413	22.1	356	634	183	0.978	98.6
27138	EModelX(+AF)	0.922	95.2	99.956	50.9	1345	1281	1200	0.972	95.6
27138	MICA	0.934	96.6	100.37	62.5	1331	1281	1204	0.981	97.1
27138	ModelAngelo	0.347	91.8	81.767	40.3	1141	1281	446	0.993	98.3
27252	EModelX(+AF)	0.875	91.7	98.2	33.9	1405	1312	1172	0.963	94.1
27252	MICA	0.888	91.2	84.527	34.7	1216	1312	1187	0.965	94.6
27252	ModelAngelo	0.31	79.4	61.123	29.3	1010	1312	409	0.968	96.7
27253	EModelX(+AF)	0.838	87	93.91	22.3	1658	1536	1321	0.935	89.3
27253	MICA	0.84	90.8	127.569	22.1	2158	1536	1324	0.968	89.7
27253	ModelAngelo	0.272	53.9	29.477	16.6	840	1536	419	0.947	93.5
27320	EModelX(+AF)	0.849	95.2	89.438	37.7	714	760	657	0.948	93.4
27320	MICA	0.874	97.9	94.68	43.9	735	760	672	0.985	95.8
27320	ModelAngelo	0.374	75.5	50.764	23.1	511	760	286	0.948	94.3
27574	EModelX(+AF)	0.994	100	100.207	241.5	1936	1932	1923	0.989	99
27574	MICA	0.998	100	100.362	241.5	1939	1932	1931	0.996	99.4
27574	ModelAngelo	0.995	99.6	99.291	481	1926	1932	1925	0.996	100
27645	EModelX(+AF)	0.959	95.3	93.793	32	1058	1075	1050	0.968	94.5
27645	MICA	0.967	95.4	94.158	33.1	1061	1075	1056	0.986	95.3
27645	ModelAngelo	0.915	91.6	84.528	49.2	992	1075	988	0.984	98.5
27656	EModelX(+AF)	0.99	99.2	109.364	51	850	771	770	1	99.2
27656	MICA	0.994	99.6	107.868	51.2	835	771	771	1	98.4
27656	ModelAngelo	0.963	96.8	98.683	39.3	786	771	749	0.969	99.1
27661	EModelX(+AF)	0.992	99.3	98.705	207	1658	1668	1658	0.996	99.9
27661	MICA	0.995	99.8	99.381	166.4	1661	1668	1661	0.994	99.6
27661	ModelAngelo	0.994	99.6	99.122	166.2	1660	1668	1658	1	100
27755	EModelX(+AF)	0.946	80.4	86.089	8.1	454	424	413	0.935	79.2
27755	MICA	0.979	84.4	89.177	9.7	448	424	421	0.952	79.9
27755	ModelAngelo	0.446	77.1	67.281	10.9	370	424	191	1	86.9
27758	EModelX(+AF)	0.396	47.5	29.889	10.7	1373	2182	939	0.784	61.1
27758	MICA	0.601	66	53.447	14.7	1767	2182	1347	0.952	84.6
27758	ModelAngelo	0.22	25.1	6.407	19.6	557	2182	488	0.893	96.9
27760	EModelX(+AF)	0.391	51.5	33.457	10.4	1628	2506	1093	0.625	58.4
27760	MICA	0.459	65.9	46.703	20.9	1776	2506	1192	0.898	90.6
27760	ModelAngelo	0.311	34.4	12.025	18.3	876	2506	784	0.963	94.9
27761	EModelX(+AF)	0.309	34.4	15.599	11.7	945	2084	690	0.806	72.3
27761	MICA	0.441	67.4	54.043	15.8	1671	2084	953	0.962	86.5
27761	ModelAngelo	0.168	19.6	3.922	17.7	417	2084	358	0.95	96.8
27899	EModelX(+AF)	0.985	97.7	102.202	30.3	681	651	649	0.982	95.9
27899	MICA	0.989	98.8	104.719	58.5	690	651	651	0.983	98
27899	ModelAngelo	0.459	92	85.641	37.4	606	651	301	0.99	98.8
28064	EModelX(+AF)	0.977	98.9	109.496	68.4	2263	2044	2006	0.983	97.1
28064	MICA	0.977	99.1	109.475	86.5	2258	2044	2004	0.981	97.8
28064	ModelAngelo	0.53	95.3	95.626	53.1	2051	2044	1094	0.953	97.8
28065	EModelX(+AF)	0.905	93.5	87.302	79.9	2296	2459	2240	0.979	97.2
28065	MICA	0.917	93.8	87.735	118.1	2300	2459	2262	0.993	99.2
28065	ModelAngelo	0.823	93.9	87.179	124.8	2283	2459	2025	0.997	99.5
28641	EModelX(+AF)	0.984	98.5	120.567	101.1	1885	1540	1521	0.993	98.2
28641	MICA	0.995	99.5	117.139	127.7	1813	1540	1537	0.995	99.1
28641	ModelAngelo	0.958	96.4	94.772	92.8	1514	1540	1477	0.998	99.8
28660	EModelX(+AF)	0.496	76.6	83.221	6.5	641	590	344	0.689	44.5
28660	MICA	0.647	88.1	102.435	9.5	686	590	411	0.946	73.7
28660	ModelAngelo	0.092	8.8	0.82	8.7	55	590	55	0.982	92.3
28666	EModelX(+AF)	0.924	95.9	89.396	152	591	634	591	1	98.4

28666	MICA	0.846	94	89.849	37.2	606	634	545	0.989	95.3
28666	ModelAngelo	0.926	92.6	86.174	48.9	590	634	590	0.983	98
28866	EModelX(+AF)	0.592	59.6	45.403	9.8	4215	5533	3462	0.826	65.9
28866	MICA	0.611	62.5	51.701	9.6	4577	5533	3630	0.769	63.6
28866	ModelAngelo	0.162	29.5	8.883	16.2	1666	5533	899	0.973	90
28867	EModelX(+AF)	0.611	60.5	43.464	11.4	3605	5018	3223	0.818	72.4
28867	MICA	0.588	65	51.101	9.9	3945	5018	3110	0.779	63.8
28867	ModelAngelo	0.188	38.2	14.883	14.5	1955	5018	947	0.975	88.2
33187	EModelX(+AF)	0.888	88.8	93.191	44.1	573	546	497	0.966	95.9
33187	MICA	0.976	96.9	113.227	35.3	638	546	540	0.98	96.4
33187	ModelAngelo	0.694	91.6	123.643	41.7	737	546	381	0.99	98.4
33233	EModelX(+AF)	0.971	97.2	98.36	42.4	1102	1089	1070	0.98	96.5
33233	MICA	0.983	98.8	102.792	63.3	1133	1089	1079	0.994	97.6
33233	ModelAngelo	0.914	91.3	83.755	35.5	999	1089	999	0.993	99.5
33242	EModelX(+AF)	0.985	98.4	97.777	109.6	1882	1894	1874	0.999	98.8
33242	MICA	0.991	99	99.366	134	1901	1894	1883	0.997	99
33242	ModelAngelo	0.938	94.1	88.983	63.6	1791	1894	1779	0.993	98.7
33243	EModelX(+AF)	0.994	98.8	98.852	93.5	1895	1894	1891	1	98.7
33243	MICA	0.996	99.2	100.038	110.5	1910	1894	1893	0.995	98.9
33243	ModelAngelo	0.881	93.8	88.649	65.8	1790	1894	1670	0.996	99.3
33244	EModelX(+AF)	0.994	98.7	98.856	77.9	1897	1894	1891	0.999	98.8
33244	MICA	0.988	98.1	98.618	61.9	1904	1894	1882	0.989	97.5
33244	ModelAngelo	0.841	88.3	78.556	76	1685	1894	1595	0.999	99.2
33245	EModelX(+AF)	0.995	98.7	99.06	99.7	1926	1919	1917	0.999	98.6
33245	MICA	0.973	97.2	96.238	77.8	1900	1919	1874	1	98.1
33245	ModelAngelo	0.924	93.9	88.42	112.6	1807	1919	1774	0.997	99.7
33331	EModelX(+AF)	0.982	97.6	97.067	57.7	1822	1832	1811	0.991	97.1
33331	MICA	0.987	98.7	98.323	56.5	1825	1832	1816	0.993	97.8
33331	ModelAngelo	0.944	96.9	94.15	49.3	1780	1832	1734	0.991	96.6
33348	EModelX(+AF)	0.993	98.5	98.876	50.1	1839	1832	1829	0.995	96.7
33348	MICA	0.993	98.5	98.5	60.1	1832	1832	1828	0.997	97.4
33348	ModelAngelo	0.964	97	95.2	55.5	1798	1832	1773	0.994	97.6
33430	EModelX(+AF)	0.902	93.8	86.486	62.7	1218	1321	1203	0.984	96
33430	MICA	0.926	95.8	91.014	58	1255	1321	1231	0.972	96.1
33430	ModelAngelo	0.356	94.1	85.979	74.8	1207	1321	471	1	98.9
33431	EModelX(+AF)	0.921	93.3	87.332	43.7	1873	2001	1865	0.959	95.5
33431	MICA	0.939	95.9	92.593	60.9	1932	2001	1898	0.992	97.5
33431	ModelAngelo	0.469	95.7	90.822	60.7	1899	2001	939	0.999	99.2
33432	EModelX(+AF)	0.904	92.4	85.557	54.9	1863	2012	1833	0.981	96.9
33432	MICA	0.949	97.8	94.932	53.3	1953	2012	1920	0.981	95.2
33432	ModelAngelo	0.465	95.6	89.993	62.5	1894	2012	937	0.984	98.3
33433	EModelX(+AF)	0.927	95.3	90.239	56.1	1890	1996	1860	0.981	96.2
33433	MICA	0.931	96	91.094	49.1	1894	1996	1869	0.989	96.1
33433	ModelAngelo	0.455	94.9	87.911	49.8	1849	1996	911	0.968	98.8
33439	EModelX(+AF)	0.938	96.1	97.353	51.8	2020	1994	1887	0.976	96
33439	MICA	0.944	97	93.789	53.7	1928	1994	1895	0.978	96.6
33439	ModelAngelo	0.445	93.6	85.526	53.4	1822	1994	889	0.988	98.6
33528	EModelX(+AF)	0.409	63.7	44.964	9.2	3367	4770	2062	0.799	54.9
33528	MICA	0.601	75.2	61.626	14.7	3909	4770	2955	0.9	72.8
33528	ModelAngelo	0.56	82.7	69.454	36.5	4006	4770	2682	0.964	94.6
33676	EModelX(+AF)	0.942	98.7	100.354	41.4	1274	1253	1189	0.985	96.2
33676	MICA	0.951	98.4	101.62	57	1294	1253	1203	0.983	97.3
33676	ModelAngelo	0.473	98.9	97.953	86.1	1241	1253	594	0.998	99.3
33677	EModelX(+AF)	0.935	98.1	98.33	70.6	1281	1278	1204	0.978	97.5
33677	MICA	0.949	99.4	97.222	86.9	1250	1278	1217	0.985	97.7
33677	ModelAngelo	0.551	99.2	96.716	110.3	1246	1278	706	0.992	99.8

33678	EModelX(+AF)	0.895	94.4	101.579	38.4	1514	1407	1277	0.964	95.1
33678	MICA	0.922	97.9	108.337	50.5	1557	1407	1303	0.989	97
33678	ModelAngelo	0.324	97.6	98.363	87.3	1418	1407	456	1	99.8
33853	EModelX(+AF)	0.77	83.1	79.561	18.2	607	634	510	0.898	82.5
33853	MICA	0.836	91.5	91.644	30.5	635	634	548	0.927	86
33853	ModelAngelo	0.667	82.3	69.968	18.6	539	634	431	0.937	94.3
33854	EModelX(+AF)	0.823	88.3	87.186	20	626	634	535	0.907	83.4
33854	MICA	0.825	92.9	95.538	18.4	652	634	547	0.874	80.8
33854	ModelAngelo	0.707	90.2	83.513	21.2	587	634	453	0.965	93.4
33955	EModelX(+AF)	0.921	92.7	88.399	31.9	1480	1552	1455	0.961	93.4
33955	MICA	0.934	94	90.184	35.6	1489	1552	1471	0.959	95.4
33955	ModelAngelo	0.393	85.2	71.75	46.1	1307	1552	612	0.993	98.8
33956	EModelX(+AF)	0.943	97.3	93.284	53.4	1533	1599	1519	0.99	97.9
33956	MICA	0.945	97.1	93.456	53.2	1539	1599	1524	0.988	97.5
33956	ModelAngelo	0.399	91	79.789	73.6	1402	1599	639	0.986	98.8
33957	EModelX(+AF)	0.898	93.7	86.155	57.6	1473	1602	1463	0.948	95
33957	MICA	0.946	98.2	94.093	75.4	1535	1602	1525	0.996	98.6
33957	ModelAngelo	0.726	96.9	90.488	93.1	1496	1602	1166	0.988	99.1
33958	EModelX(+AF)	0.927	96.7	91.931	70.8	1523	1602	1497	0.986	97.4
33958	MICA	0.947	97.9	94.111	75.2	1540	1602	1527	0.996	97.8
33958	ModelAngelo	0.724	96.9	90.488	124	1496	1602	1162	0.991	99.3
33959	EModelX(+AF)	0.937	94	90.551	49.7	2074	2153	2039	0.985	95.9
33959	MICA	0.937	94	90.813	60.2	2080	2153	2042	0.972	96.8
33959	ModelAngelo	0.61	93.2	86.75	75.8	2004	2153	1316	0.995	99.2
34017	EModelX(+AF)	0.857	85.4	79.458	13.5	1150	1236	1122	0.931	81.4
34017	MICA	0.976	96.4	95.152	28.4	1220	1236	1220	0.999	95.7
34017	ModelAngelo	0.554	76.9	55.746	21.1	896	1236	689	0.984	95.6
34023	EModelX(+AF)	0.988	99	101.071	98	1415	1386	1377	0.975	98.5
34023	MICA	0.99	99.1	101.174	65.4	1415	1386	1380	0.978	97.5
34023	ModelAngelo	0.709	98.5	95.87	97.5	1349	1386	985	0.909	100
34024	EModelX(+AF)	0.982	97.6	103.764	51.9	1414	1330	1312	0.978	97.3
34024	MICA	0.98	97.5	102.558	51.9	1399	1330	1311	0.972	97.4
34024	ModelAngelo	0.67	95	92	57.5	1288	1330	896	0.985	99.4
34158	EModelX(+AF)	0.924	97.1	96.708	33	1232	1237	1159	0.949	94.9
34158	MICA	0.923	96.8	96.8	33.8	1237	1237	1155	0.952	95
34158	ModelAngelo	0.473	98.8	97.762	45.2	1224	1237	586	0.981	97.6
34270	EModelX(+AF)	0.949	97	101.143	25.5	2002	1920	1845	0.958	92.2
34270	MICA	0.954	98	100.552	34.6	1970	1920	1848	0.983	94.4
34270	ModelAngelo	0.453	91.1	83.603	28.4	1762	1920	875	0.979	96.1
34738	EModelX(+AF)	0.733	82.6	72.498	25.2	2842	3238	2389	0.989	90.8
34738	MICA	0.75	81.9	71.757	30.1	2837	3238	2445	0.987	92.5
34738	ModelAngelo	0.446	65.4	42.779	32.1	2118	3238	1447	0.991	98.1

Supplementary Table S3: Performance of MICA on 12 randomly selected EMDB cryo-EM density maps released after January 1, 2025

EMD ID	PDB ID	Resolution (Å)	TM-Score	Cα Match	Cα Quality Score	Model Length	Reference Length	Aligned Length	Sequence Identity	Sequence Match
39164	8yd4	3.69	0.772	94	90.141	2336	2436	1916	0.748	85.3
39909	8zbs	2.96	0.927	91.4	88.398	1178	1218	1159	0.959	95
44305	9b7g	2.61	0.962	98.9	126.701	2183	1704	1654	0.977	97.9
45815	9cqm	2.5	0.998	100	100	568	568	568	1	100
45823	9cqu	2.72	0.996	99.6	99.425	567	568	567	1	100
45829	9cr0	2.08	0.999	100	100.551	2009	1998	1998	0.999	100
46448	9d0a	3.1	0.932	93.4	93.314	1082	1083	1026	0.968	95.9
48384	9mm1	2.08	0.999	99.8	100.399	2010	1998	1998	1	100
61231	9j8m	3.82	0.615	82.1	62.953	1085	1415	888	0.962	94.9
62223	9kbf	3.74	0.99	99.3	100.871	578	569	569	0.995	98.8
63356	9lsi	3.3	0.979	97.7	99.52	2023	1986	1957	0.991	96.2
63358	9lsk	2.9	0.985	98.5	98.156	855	858	850	0.999	99.1
Average		2.96	0.93	96.23	96.702	1372.83	1366.75	1262.5	0.967	96.93

Supplementary Table S4: Performance metrics of MICA against ModelAngelo and EModelX(+AF) on low resolution test dataset

EMD ID	Model Type	PDB ID	Resolution (Å)	TM-Score	Cα Match	Cα Quality Score	Model Length	Reference Length	Aligned Cα Length	Sequence Identity	Sequence Match
62354	MICA	9kht	4.85	0.789	72.9	60.195	2260	2737	2222	0.939	87.3
62354	EModelX(+AF)	9kht	4.85	0.611	53.3	35.072	1801	2737	1759	0.871	74.7
62354	ModelAngelo	9kht	4.85	0.114	21.3	4.669	600	2737	318	0.833	87.3
49996	MICA	9o13	5.8	0.897	85.4	136.689	1671	1044	983	0.893	75.3
49996	EModelX(+AF)	9o13	5.8	Model Building Failed							
49996	ModelAngelo	9o13	5.8	Model Building Failed							
44981	MICA	9bwv	5.1	0.717	69.3	60.348	1773	2036	1560	0.851	74.7
44981	EModelX(+AF)	9bwv	5.1	Model Building Failed							
44981	ModelAngelo	9bwv	5.1	Model Building Failed							
45739	MICA	9cm5	4.61	0.58	52.9	43.621	1589	1927	1390	0.668	29.6
45739	EModelX(+AF)	9cm6	4.61	0.175	19.6	5.411	532	1927	407	0.334	11.6
45739	ModelAngelo	9cm7	4.61	0.002	0.7	0.005	15	1927	4	0	0
43679	MICA	8vyv	5.86	0.268	35.2	15.421	276	630	193	0.596	56.3
43679	EModelX(+AF)	8vyv	5.86	Model Building Failed							
43679	ModelAngelo	8vyv	5.86	0.019	1.7	0.032	12	630	12	0.083	0
46873	MICA	9dhq	4.78	0.629	77.8	56.880	1702	2328	1523	0.909	84.1
46873	EModelX(+AF)	9dhq	4.78	0.556	63	41.811	1545	2328	1343	0.902	71.6
46873	ModelAngelo	9dhq	4.78	0.05	20.4	4.232	483	2328	132	0.515	81.5
51169	MICA	9ga2	4.9	0.484	75.7	86.804	1540	1343	730	0.804	60.3
51169	EModelX(+AF)	9ga2	4.9	Model Building Failed							
51169	ModelAngelo	9ga2	4.9	Model Building Failed							
61490	MICA	9jhs	5.02	0.691	75.8	68.163	1446	1608	1255	0.72	69.3
61490	EModelX(+AF)	9jhs	5.02	Model Building Failed							
61490	ModelAngelo	9jhs	5.02	0.002	0.1	0.000	4	1608	4	0.25	0

Supplementary Table S5: Execution time for different protein size

EMD ID	PDB ID	Original Map Shape	Normalized Map Shape	Number of Residues	Number of Domains	AF3 Results Processing Time (min)	Domains Docking Time (min)	Atomic Model Building Time (min)	Total Time (min)
26993	8ctk	288x288x288	209x209x209	376	4	0.16	14.83	1.81	16.8
14716	7zh0	350x350x350	231x231x231	495	3	0.1	9.13	1.4	10.63
28660	8exr	300x300x300	231x231x231	590	11	0.3	189.48	5.85	195.63
15673	8aur	280x280x280	306x306x306	699	3	0.26	26.58	2.4	29.24
27656	8dql	300x300x300	330x330x330	771	9	0.26	82.55	3.56	86.37
14066	7qla	180x180x180	196x196x196	874	6	0.2	30.58	2.58	33.36
26978	8ct2	344x344x344	143x143x143	984	7	0.33	56.68	1.08	58.09
33233	7xjp	240x240x240	260x260x260	1089	6	0.2	25.36	3.01	28.57
26974	8csx	264x264x264	110x110x110	1158	6	0.26	80.4	1.95	82.61
27138	8d1v	300x300x300	249x249x249	1281	5	0.15	64.78	3.31	68.24
34023	7yqc	240x240x240	250x250x250	1386	7	0.6	26.6	1.93	29.13
15635	8at6	320x320x320	352x352x352	1580	8	0.53	32.81	5.26	38.6
14842	7zny	224x224x224	289x289x289	1879	11	0.4	153.9	6.78	161.08
27761	8dwv	300x300x300	423x423x423	2084	12	0.55	175.46	9.8	185.81
15540	8ane	350x350x350	353x353x353	2278	17	0.61	246.23	6.11	252.95
27760	8dwu	270x270x270	350x350x350	2506	19	0.81	234.35	6.93	242.09
15378	8ae1	320x320x320	349x349x349	2859	12	0.36	294.01	9.16	303.53
34738	8hgg	256x256x256	269x269x269	3238	12	0.48	201.93	9.05	211.46

Supplementary Table S6: Description of Training and Validation Dataset

EMD ID	PDB ID	Title	Contour Level	Resolution	Deposition Date	Release Date
13940	7qet	human Connexin 26 dodecamer at 20mmHg PCO2, pH7.4	0.0066	2.1	2021-12-03	2022-03-30
27672	8dr6	Closed state of RFC:PCNA bound to a nicked dsDNA	0.2	2.39	2022-07-20	2022-08-24
22833	7ke7	SARS-CoV-2 D614G 3-RBD-down Spike Protein Trimer without the P986-P987 stabilizing mutations (S-GSAS-D614G Sub-Classification)	0.2	3.32	2020-10-10	2020-11-04
31168	7eki	human alpha 7 nicotinic acetylcholine receptor in apo-form	0.28	3.18	2021-04-05	2021-05-19
33395	7xqg	Hemichannel-focused structure of C-terminal truncated connexin43/Cx43/GJA1 gap junction intercellular channel in POPE nanodiscs (GCN conformation)	0.006	3.8	2022-05-07	2023-01-25
23895	7mki	Cryo-EM structure of Escherichia coli RNA polymerase bound to lambda PR (-5G to C) promoter DNA	0.023	3.5	2021-04-23	2021-09-29
20576	6q2o	Cryo-EM structure of RET/GFRa2/NRTN extracellular complex. The 3D refinement was applied with C2 symmetry.	0.02	3.65	2019-08-08	2019-10-02
22362	7jk5	Structure of Drosophila ORC bound to DNA	0.006	3.9	2020-07-27	2020-09-09
22822	7kdh	SARS-CoV-2 RBD up Spike Protein Trimer without the P986-P987 stabilizing mutations (S-GSAS)	0.25	3.33	2020-10-08	2020-11-04
31950	7vfe	Cryo-EM structure of Vaccinia virus scaffolding protein D13 with N-terminal polyhistidine tag	5	2.63	2021-09-13	2022-02-23
23827	7mge	Structure of C9orf72:SMCR8:WDR41 in complex with ARF1	0.27	3.94	2021-04-12	2021-06-23
30784	7dni	MDA5 CARDs-MAVS CARD polyUb complex	0.513	3.2	2020-12-09	2021-10-13
23897	7mkj	Cryo-EM structure of Escherichia coli RNA polymerase bound to T7A1 promoter DNA	0.34	2.9	2021-04-23	2021-09-29
13068	7otw	DNA-PKcs in complex with AZD7648	0.2	2.99	2021-06-10	2022-01-12
25423	7stb	Closed state of Rad24-RFC:9-1-1 bound to a 5' ss/dsDNA junction	0.25	2.72	2021-11-12	2022-03-23
32049	7vo9	Streptomyces coelicolor zinc uptake regulator complexed with zinc and DNA (dimer of dimers)	0.47	3.8	2021-10-13	2022-08-03
15417	8ag6	human MutSalpha (MSH2/MSH6) binding to DNA with a GT mismatch	5.5	2.8	2022-07-19	2023-01-25
25745	7t8t	CryoEM structure of PLCg1	0.07	3.68	2021-12-17	2022-12-21
32852	7wvf	ectoTLR3-mAb12-poly(I:C) complex	0.7	3.91	2022-02-10	2022-11-16
23213	7l7i	Cryo-EM structure of Hsp90:FKBP51:p23 closed-state complex	0.25	3.3	2020-12-28	2021-08-25
26567	7ujn	Structure of Human SAMHD1 with Non-Hydrolysable dGTP Analog	0.025	2.89	2022-03-31	2022-07-20
10095	6s3r	Structure of the FliPQR complex from the flagellar type 3 secretion system of Pseudomonas savastanoi.	0.0166	3.5	2019-06-25	2020-03-25
27893	8e4n	The closed C1-state mouse TRPM8 structure in complex with PI(4,5)P2	0.14	3.07	2022-08-18	2022-10-26
11332	6zp0	Structure of SARS-CoV-2 Spike Protein Trimer (single Arg S1/S2 cleavage site) in Closed State	0.0222	3.0	2020-07-08	2020-07-22
30028	6m02	cryo-EM structure of human Pannexin 1 channel	0.0285	3.2	2020-02-19	2020-03-25
15914	8b8j	Cryo-EM structure of Ca2+-bound mTMEM16F F518H mutant in Digitonin	0.149	2.96	2022-10-04	2022-11-16
25363	7soy	The structure of the PP2A-B56gamma1 holoenzyme-PME-1 complex	0.04	3.4	2021-11-01	2022-08-31
26540	7ui6	CryoEM structure of LARGE1 from C1 reconstruction	0.012	3.7	2022-03-28	2023-03-08
27285	8dbi	Human PRPS1 with Phosphate, ATP, and R5P; Hexamer	1.25	2.0	2022-06-14	2023-02-15
26128	7tu7	Structure of the L. blandensis dGTPase H125A mutant bound to dGTP	0.1	2.5	2022-02-02	2022-06-01
25986	7tlb	Down-state locked rS2d SARS-CoV-2 spike ectodomain in the RBD-down conformation, State 2	0.432	3.06	2022-01-18	2022-02-02

32357	7w8j	Dimethylformamidase, 2x(A2B2)	0.09	2.5	2021-12-07	2022-04-06
23722	7m9b	ADP-AlF3 bound TnsC structure in closed form	0.0078	3.8	2021-03-30	2021-07-28
26156	7tx7	Cryo-EM structure of the human reduced folate carrier	0.2	3.8	2022-02-07	2022-09-21
4219	6fay	Teneurin3 monomer	0.4	3.8	2017-12-18	2018-03-28
33337	7xo5	SARS-CoV-2 Omicron BA.1 Variant Spike Trimer with one mouse ACE2 Bound	0.03	3.13	2022-05-01	2022-06-15
20860	6urg	Cryo-EM structure of human CPSF160-WDR33-CPSF30-CPSF100 PIM complex	0.0315	3.0	2019-10-23	2019-11-27
25226	7snh	Structure of G6PD-D200N tetramer bound to NADP+	0.4	2.2	2021-10-28	2022-07-13
26480	7uft	Cryo-EM Structure of Bl_Man38C at 2.9 A	4	2.9	2022-03-23	2022-11-16
24486	7rje	Complex III2 from Candida albicans, Inz-5 bound	0.52	3.3	2021-07-20	2021-09-15
31912	7vda	2.26 A structure of the glutamate dehydrogenase	0.013	2.26	2021-09-06	2021-12-29
7348	6c6l	Yeast Vacuolar ATPase Vo in lipid nanodisc	0.035	3.5	2018-01-19	2018-03-21
26356	7u5n	Cryo-EM Structure of Glutamine Synthetase	0.1	2.58	2022-03-02	2022-12-14
20152	6opp	Asymmetric reconstruction of CD4- and 17-bound B41 HIV-1 Env SOSIP in complex with DDM	0.58	3.7	2019-04-25	2020-10-21
13256	7p8w	Human erythrocyte catalase cryoEM	0.055	2.2	2021-07-23	2021-08-25
24439	7rfe	HUMAN IMPDH1 TREATED WITH GTP, IMP, AND NAD+; INTERFACE-CENTERED	0.0605	2.6	2021-07-14	2022-01-12
26047	7tp7	Delta (B.1.617.2) SARS-CoV-2 variant spike protein (S-GSAS-Delta) in the 1-RBD-up conformation; Subclassification D11 state	0.8	3.48	2022-01-25	2022-02-09
13937	7qeq	human Connexin 26 dodecamer at 90mmHg PCO2, pH7.4	0.0066	1.9	2021-12-03	2022-03-30
20354	6pk4	cryoEM structure of the substrate-bound human CTP synthase 2 filament	4.4	3.5	2019-06-28	2019-12-25
22972	7kod	Cryo-EM structure of heavy chain mouse apoferritin	0.007	1.655	2020-11-08	2020-12-16
28879	8f6c	E. coli cytochrome bo3 ubiquinol oxidase dimer	0.095	3.46	2022-11-16	2022-11-30
13076	7oug	STLV-1 intasome:B56 in complex with the strand-transfer inhibitor raltegravir	0.0187	3.1	2021-06-11	2021-08-18
21407	6vvy	Mycobacterium tuberculosis WT RNAP transcription open promoter complex with Sorangicin	0.433	3.42	2020-02-18	2020-10-21
20541	6pzz	CryoEM map of NA-80 Fab in complex with N9 Shangahi2	0.641	3.6	2019-08-01	2019-12-04
14220	7r06	Abortive infection DNA polymerase AbiK from Lactococcus lactis	2.5	2.27	2022-02-01	2022-09-07
11204	6zgf	Spike Protein of RaTG13 Bat Coronavirus in Closed Conformation	0.7	3.1	2020-06-18	2020-07-01
30555	7d3e	Cryo-EM structure of human DUOX1-DUOXA1 in low-calcium state	0.02	2.8	2020-09-19	2020-12-09
11150	6zbc	Merozoite surface protein 1 (MSP-1) from Plasmodium falciparum, main conformation	0.3	3.1	2020-06-08	2021-05-19
22835	7ke9	SARS-CoV-2 D614G 1-RBD-up Spike Protein Trimer without the P986-P987 stabilizing mutations (S-GSAS-D614G sub-classification)	0.25	3.08	2020-10-10	2020-11-04
25756	7t9f	Structure of VcINDY-apo	4.8	3.23	2021-12-19	2022-05-25
26436	7ub6	SARS-CoV-2 Omicron-BA.2 3-RBD down Spike Protein Trimer without the P986-P987 stabilizing mutations (S-GSAS-Omicron-BA.2)	0.867	3.52	2022-03-14	2022-04-20
26625	7unk	Structure of Importin-4 bound to the H3-H4-ASF1 histone-histone chaperone complex	0.12	3.45	2022-04-11	2022-09-21
23816	7mfg	Cryo-EM structure of the VRC310 clinical trial, vaccine-elicited, human antibody 310-030-1D06 Fab in complex with an H1 NC99 HA trimer	0.12	3.87	2021-04-09	2021-11-03
31053	7ebf	Cryo-EM structure of Isocitrate lyase-1 from Candida albicans	0.0154	2.63	2021-03-09	2021-06-23
15635	8at6	Cryo-EM structure of yeast Elp456 subcomplex	0.0242	3.7	2022-08-22	2022-12-07

12665	7nzm	Cryo-EM structure of pre-dephosphorylation complex of phosphorylated eIF2alpha with trapped holophosphatase (PP1A_D64A/PPP1R15A/G-actin/DNase I)	0.05	3.96	2021-03-24	2021-09-29
23952	7mr0	Cryo-EM structure of RecBCD with docked RecBNuc and flexible RecD	0.025	3.7	2021-05-07	2021-07-28
11153	6zbf	Merozoite surface protein 1 (MSP-1) from Plasmodium falciparum, alternative conformation 3	0.3	3.2	2020-06-08	2021-05-19
30416	7cn4	Cryo-EM structure of bat RaTG13 spike glycoprotein	0.0082	2.93	2020-07-30	2021-03-03
25873	7tfi	Cryo-EM 3D map of the S. cerevisiae clamp-clamp loader complex PCNA-RFC bound to DNA with an open clamp	0.2	3.41	2022-01-06	2022-11-16
28560	8ert	NLRP3 PYD filament	0.4	3.3	2022-10-12	2022-12-14
23971	7msx	SARS-CoV-2 Nsp2	0.032	3.15	2021-05-12	2021-05-26
6714	5xb1	human ferritin mutant - E-helix deletion	0.3	3.0	2017-03-15	2018-02-21
14117	7qpc	Inward-facing NPA bound form of auxin transporter PIN8	0.28	3.44	2022-01-03	2022-07-06
21409	6vw0	Mycobacterium tuberculosis RNAP S456L mutant open promoter complex	0.387	3.59	2020-02-18	2020-10-21
23520	7luc	Cryo-EM structure of RSV preF bound by Fabs 32.4K and 01.4B	0.127	3.21	2021-02-22	2021-04-21
23135	7l2o	Cryo-EM structure of RTX-bound full-length TRPV1 at pH 5.5	0.008	3.64	2020-12-17	2021-09-22
23638	7m2z	Monomeric single-particle reconstruction of the Yeast gamma-TuSC	2.5	3.7	2021-03-17	2021-05-12
26126	7tu5	Structure of the L. blandensis dGTPase in the apo form	0.0295	2.1	2022-02-02	2022-06-01
0828	6l42	Structure of severe fever with thrombocytopenia syndrome virus L protein	0.0085	3.4	2019-10-15	2020-05-13
27025	8cwl	CRYO-EM STRUCTURE OF HUMAN 15-PGDH IN COMPLEX WITH SMALL MOLECULE SW222746	0.15	2.9	2022-05-19	2023-03-08
15527	8amw	AQP7 dimer of tetramers_C1	0.2	3.0	2022-08-04	2023-02-15
12701	7oqh	CryoEM structure of the transcription termination factor Rho from Mycrobacterium Tuberculosis	0.0344	3.32	2021-04-01	2022-02-23
21461	6vyi	Cryo-EM structure of human diacylglycerol O-acyltransferase 1	0.069	3.0	2020-02-26	2020-05-13
31911	7vd9	2.29 A structure of the human catalase	0.02	2.29	2021-09-06	2021-12-29
21991	6x17	Outward-facing state of the glutamate transporter homologue GltPh in complex with TBOA	0.0328	3.66	2020-05-18	2020-11-18
9590	6acf	structure of leucine dehydrogenase from Geobacillus stearothermophilus by cryo-EM	0.05	3.0	2018-07-26	2018-12-26
24078	7my3	CryoEM structure of neutralizing nanobody Nb12 in complex with SARS-CoV2 spike	0.3	2.9	2021-05-20	2021-06-16
26129	7tu8	Structure of the L. blandensis dGTPase H125A mutant bound to dGTP and dATP	0.106	2.6	2022-02-02	2022-06-01
22137	6xdg	Complex of SARS-CoV-2 receptor binding domain with the Fab fragments of two neutralizing antibodies	0.1	3.9	2020-06-10	2020-06-24
24454	7rgq	HUMAN RETINAL VARIANT IMPDH1(546) TREATED WITH GTP, ATP, IMP, NAD+; INTERFACE-CENTERED	2.39	3.9	2021-07-15	2022-01-12
33300	7xml	Cryo-EM structure of PEIP-Bs_enolase complex	0.6	3.2	2022-04-26	2022-07-27
21383	6vtt	Cryo-EM Structure of CAP256-VRC26.25 Fab bound to HIV-1 Env trimer CAP256.wk34.c80 SOSIP.RnS2	0.4	3.7	2020-02-13	2020-04-08
23146	7l2z	Bacterial cellulose synthase BcsB hexamer	0.3	3.4	2020-12-17	2021-03-24
24524	7rlb	Cryo-EM structure of human p97-A232E mutant bound to ADP	0.1	3.3	2021-07-23	2021-09-22
24794	7s15	GLP-1 receptor bound with Pfizer small molecule agonist	4.2	3.8	2021-09-01	2022-06-08
13467	7pkb	C-reactive protein pentamer at pH 7.5	0.0151	3.2	2021-08-25	2021-12-22
31715	7v4l	Cryo-EM Structure of Camellia sinensis glutamine synthetase CsGSIb inactive Pentamer State III	0.065	3.4	2021-08-13	2022-05-18
23706	7m6s	Full length alpha1 Glycine receptor in presence of 1mM Glycine and 32uM Tetrahydrocannabinol State 3	0.009	3.61	2021-03-26	2022-08-03

32591	7wlr	Cryo-EM structure of the nucleosome containing Komagataella pastoris histones	3.94	3.54	2022-01-13	2022-07-13
32568	7wks	Apo state of AtPIN3	0.0179	3.0	2022-01-11	2022-08-10
27286	8dbj	Human PRPS1 with Phosphate, ATP, and R5P; Filament Interface	1.25	2.0	2022-06-14	2023-02-15
22456	7jsj	Structure of the NaCT-PF2 complex	10.1	3.12	2020-08-14	2021-02-24
31490	7f8n	Human pannexin-1 showing a conformational change in the N-terminal domain and blocked pore	0.015	3.4	2021-07-02	2022-01-26
21593	6wbm	Cryo-EM structure of human Pannexin 1 channel N255A mutant	0.0075	2.86	2020-03-26	2020-06-03
27394	8dep	Cryo-EM structure of the human reduced folate carrier, apo condition	0.1	3.6	2022-06-21	2022-09-21
30587	7d61	Cryo-EM Structure of human CALHM5 in the presence of EDTA	0.019	2.8	2020-09-28	2020-12-23
3699	5nv3	Structure of Rubisco from Rhodobacter spheroides in complex with CABP	0.9	3.39	2017-05-03	2017-07-26
15804	8b1t	RecBCD-DNA in complex with the phage protein Abc2	0.016	3.4	2022-09-12	2022-12-28
12739	7o6p	Structure of the borneol dehydrogenase 2 of Salvia officinalis	0.5	2.04	2021-04-12	2021-12-01
23815	7mff	Dimeric (B-Raf)2:(14-3-3)2 complex bound to SB590885 Inhibitor	0.049	3.89	2021-04-09	2022-01-26
27287	8dbk	Human PRPS1 with Phosphate, ATP, and R5P; Hexamer with resolved catalytic loops	1.25	2.1	2022-06-14	2023-02-15
26481	7ufu	Cryo-EM Structure of Bl_Man38A nucleophile mutant in complex with mannose at 2.7 A	4.5	2.7	2022-03-23	2022-11-16
14445	7z19	E. coli C-P lyase bound to a single PhnK ABC domain	0.25	2.57	2022-02-24	2022-05-25
31048	7eb0	Cryo-EM structure of SARS-CoV-2 Spike D614G variant, one RBD-up conformation 2	0.22	3.6	2021-03-08	2021-06-23
14116	7qpa	Outward-facing auxin bound form of auxin transporter PIN8	0.24	3.18	2022-01-03	2022-07-06
24700	7ru9	Metazoan pre-targeting GET complex (cBUGG-in)	0.02	3.3	2021-08-16	2021-12-15
34034	7yqt	SARS-CoV-2 BA.2.75 S Trimer (1 RBD Up)	0.4	3.45	2022-08-08	2022-10-19
21406	6vvx	Mycobacterium tuberculosis WT RNAP transcription initiation intermediate structure with Sorangicin	0.48	3.39	2020-02-18	2020-10-21
26355	7u5m	Cryo-EM Structure of GAPDH	0.15	2.28	2022-03-02	2022-12-14
25413	7sss	Structure of the NADH-bound human COQ7:COQ9 complex by single-particle electron cryo-microscopy	0.035	2.4	2021-11-11	2022-11-02
4608	6qnt	Human Adenovirus type 3 fiber knob in complex with one copy of Desmoglein-2	0.07	3.5	2019-02-12	2019-03-27
32773	7wta	Cryo-EM structure of human pyruvate carboxylase in apo state	0.007	3.9	2022-02-04	2022-11-09
7297	6bwd	3.7 angstrom cryoEM structure of truncated mouse TRPM7	0.007	3.7	2017-12-14	2018-08-15
9875	6jqn	Structure of PaaZ, a bifunctional enzyme in complex with NADP+ and OCoA	0.085	3.1	2019-03-31	2019-09-11
32446	7wec	SARS-CoV-2 Omicron variant spike protein with three XGv347 Fabs binding to three closed state RBDs	0.219	3.3	2021-12-23	2022-05-04
0730	6knf	CryoEM map and model of Nitrite Reductase at pH 6.2	0.0399	2.99	2019-08-05	2020-08-12
20040	6of2	Precursor ribosomal RNA processing complex, State 2.	4.5	2.9	2019-03-28	2019-09-11
22274	6xnz	Structure of RAG1 (R848M/E649V)-RAG2-DNA Target Capture Complex	0.03	3.8	2020-07-05	2020-08-26
12686	7o11	ABC transporter NosDFY, nucleotide-free in GDN, R-domain 1	0.014	3.7	2021-03-28	2022-04-13
13077	7ouh	Structure of the STLV intasome:B56 complex bound to the strand-transfer inhibitor bictegravir	0.0656	3.5	2021-06-11	2021-08-18
20153	6opq	Reconstruction of class 1 of CD4- and 17-bound B41 HIV-1 Env SOSIP incubated with LMNG and small molecule GO27	0.3	3.8	2019-04-25	2020-10-21
12687	7o12	ABC transporter NosDFY, AMPPNP-bound in GDN	0.015	3.7	2021-03-28	2022-04-13

23300	7lf6	Structure of lysosomal membrane protein	0.161	3.5	2021-01-15	2022-01-26
30115	6m67	The Cryo-EM Structure of Human Pannexin 1 with D376E/D379E Mutation	1.06	3.6	2020-03-13	2020-04-15
21042	6v4n	Structure of human 1G05 Fab in complex with influenza virus neuraminidase from B/Phuket/3073/2013	0.0152	2.5	2019-11-28	2020-10-07
14858	7zpm	Influenza A/H7N9 polymerase apo-protein dimer complex	0.2	2.81	2022-04-27	2022-12-28
31468	7f5v	Drosophila P5CS filament with glutamate, ATP, and NADPH	0.01	3.6	2021-06-22	2022-04-06
23115	7l1q	PS3 F1-ATPase Binding/TS Dwell	0.15	3.4	2020-12-15	2021-07-21
23595	7lym	South African (B.1.351) SARS-CoV-2 spike protein variant (S-GSAS-B.1.351) in the RBD-down conformation	0.18	3.57	2021-03-07	2021-03-31
10888	6yrf	Vip3Bc1 tetramer	0.83	3.9	2020-04-20	2021-03-17
32933	7x13	Structure of IgG-Fc hexamer	0.0127	3.7	2022-02-23	2023-03-01
27291	8dbn	Human PRPS1-E307A engineered mutation with Phosphate, ATP, and R5P; Hexamer	1.5	2.4	2022-06-14	2023-02-15
25558	7sz0	Cryo-EM structure of the extracellular module of the full-length EGFR L834R bound to EGF. "tips-juxtaposed" conformation	0.2	3.3	2021-11-25	2021-12-22
30035	6m0s	3.6A Yeast Vo state3 prime	0.01	3.6	2020-02-22	2020-11-04
23124	7l6o	Cryo-electron microcospy reconstruction of CH848.3.D0949.10.17chim.6R.DS.SOSIP.664 HIV Env	0.45	3.9	2020-12-16	2021-02-10
21141	6vak	Cryo-EM structure of human CALHM2	2.05	3.48	2019-12-17	2020-01-29
0827	6l3v	The R15G mutant of human Cx31.3/GJC3 connexin hemichannel	1	2.63	2019-10-15	2020-09-09
33836	7yhq	CryoEM structure of Arabidopsis ROS1 in complex with a covalent-linked reaction intermediate at 3.9 Angstroms resolution	0.00675	3.9	2022-07-14	2022-11-30
23892	7mkd	Cryo-EM structure of Escherichia coli RNA polymerase bound to lambda PR promoter DNA (class 1)	0.27	3.2	2021-04-23	2021-09-29
23806	7mex	Structure of yeast Ubr1 in complex with Ubc2 and N-degron	0.015	3.35	2021-04-08	2021-11-24
26127	7tu6	Structure of the L. blandensis dGTPase bound to dATP	0.0984	2.7	2022-02-02	2022-06-01
26443	7ucg	Structure of the DU422 SOSIP.664 trimer in complex with neutralizing antibody Fab fragments 10-1074 and BG24	0.013	3.5	2022-03-16	2022-08-17
27799	8dzi	Structure of SARS-CoV-2 Omicron BA.1.1.529 Spike trimer with one RBD down in complex with the Fab fragment of human neutralizing antibody MB.02	0.02	3.5	2022-08-07	2022-08-31
20584	6tys	A potent cross-neutralizing antibody targeting the fusion glycoprotein inhibits Nipah virus and Hendra virus infection	0.9	3.5	2019-08-09	2019-10-09
20090	6omf	CryoEM structure of SigmaS-transcription initiation complex with activator Crl	0.45	3.26	2019-04-10	2019-08-28
14115	7qp9	Outward-facing apo-form of auxin transporter PIN8	0.15	2.89	2022-01-03	2022-07-06
15805	8b1u	RecBCD-DNA in complex with the phage protein Abc2 and host PpiB	0.015	3.8	2022-09-12	2022-12-28
29714	8g46	Cryo-EM structure of DDB1deltaB-DDA1-DCAF16-BRD4(BD2)-MMH2	0.463	2.2	2023-02-08	2023-03-08
30810	7dpt	Structural basis for ligand binding modes of CTP synthase	0.1	2.48	2020-12-21	2021-09-15
15646	8atd	Wild type hexamer oxalyl-CoA synthetase (OCS)	0.027	3.1	2022-08-23	2023-02-08
30462	7cte	Human Origin Recognition Complex, ORC2-5	0.3	3.8	2020-08-18	2021-01-06
30527	7d09	Acinetobacter MlaFEDB complex in ATP-bound Vtrans2 conformation	0.03	3.6	2020-09-09	2020-12-16
10493	6tfk	Vip3Aa toxin structure	0.025	2.9	2019-11-14	2020-08-12
25170	7sk2	Human wildtype GABA reuptake transporter 1 in complex with tiagabine, inward-open conformation	0.11	3.82	2021-10-19	2022-06-08
23134	7l2n	Cryo-EM structure of RTX-bound full-length TRPV1 in C1 state	0.015	3.09	2020-12-17	2021-09-22
13938	7qer	human Connexin 26 dodecamer at 55mm Hg PCO2, pH7.4	0.0115	2.2	2021-12-03	2022-03-30
11191	6zg1	SARM1 SAM1-2 domains	0.18	3.77	2020-06-18	2020-11-11

23117	7l1s	PS3 F1-ATPase Pi-bound Dwell	0.15	3.6	2020-12-15	2021-07-21
4733	6r69	Improved map of the FliPQR complex that forms the core of the Salmonella type III secretion system export apparatus.	0.018	3.65	2019-03-26	2019-05-29
24701	7rua	Metazoan pre-targeting GET complex (cBUGG-out)	0.02	3.4	2021-08-16	2021-12-15
22829	7kdt	Human Tom70 in complex with SARS CoV2 Orf9b	0.5	3.05	2020-10-09	2020-10-21
10217	6sjg	Cryo-EM structure of the RecBCD no Chi negative control complex	0.01	3.8	2019-08-13	2020-01-01
9906	6k1h	Structure of membrane protein	0.12	3.52	2019-05-10	2019-07-10
24441	7rfg	HUMAN IMPDH1 TREATED WITH GTP, IMP, AND NAD+ OCTAMER-CENTERED	1.18	2.6	2021-07-14	2022-01-12
9873	6jql	Structure of PaaZ, a bifunctional enzyme	0.085	2.9	2019-03-31	2019-09-11
27891	8e4l	The open state mouse TRPM8 structure in complex with the cooling agonist C3, AITC, and PI(4,5)P2	0.12	3.32	2022-08-18	2022-10-26
23412	7ll2	Cryo-EM map of BG505 DS-SOSIP in complex with Glycan276-Dependent Broadly Neutralizing Antibody VRC33.01 Fab	1.28	3.73	2021-02-03	2021-03-17
23049	7kvf	Cryo-EM structure of human Factor V at 3.6 Angstrom resolution	0.2	3.6	2020-11-28	2021-03-24
16783	8cpm	Human apoferritin after 405 nm laser exposure	0.025	1.81	2023-03-03	2023-03-15
20770	6uhc	CryoEM structure of human Arp2/3 complex with bound NPFs	0.475	3.9	2019-09-27	2020-07-01
4734	6r6b	Structure of the core Shigella flexneri type III secretion system export gate complex SctRST (Spa24/Spa9/Spa29).	0.01	3.5	2019-03-26	2019-05-29
7876	6dfh	BG505 MD64 N332-GT2 SOSIP trimer in complex with germline-reverted BG18 fragment antigen binding	0.03	3.85	2018-05-14	2019-11-06
10203	6shq	Escherichia coli AGPase in complex with AMP. Symmetry C2	0.3	3.2	2019-08-07	2020-02-05
32928	7x0x	Cryo-EM Structure of Arabidopsis CRY2 in active conformation	0.8	2.56	2022-02-22	2023-01-04
22838	7kec	SARS-CoV-2 D614G 1-RBD-up Spike Protein Trimer without the P986-P987 stabilizing mutations (S-GSAS-D614G Sub-Classification)	0.2	3.84	2020-10-10	2020-11-04
0750	6kq4	323 K cryoEM structure of Sso-KARI in complex with Mg2+	1.56	2.3	2019-08-16	2020-03-25
9954	6ka4	Cryo-EM structure of the AtMLKL3 tetramer	0.025	3.4	2019-06-20	2020-09-23
26838	7uwq	Klebsiella pneumoniae adenosine monophosphate nucleosidase	0.604	3.05	2022-05-03	2022-09-28
23902	7mkp	Escherichia coli RNA polymerase core enzyme	0.23	3.41	2021-04-26	2021-06-23
23000	7kr2	ClpP from Neisseria meningitidis - Compressed conformation	1.23	3.2	2020-11-18	2021-11-24
0991	6lve	Structure of Dimethylformamidase, tetramer, E521A mutant	0.061	3.1	2020-02-02	2020-06-03
0071	6gve	GAPDH-CP12-PRK complex	0.00952	3.9	2018-06-20	2019-07-03
20993	6v0c	Lipophilic Envelope-spanning Tunnel B (LetB), Map 1	0.055	3.46	2019-11-18	2020-05-06
32351	7w84	CryoEM structure of apo form ZmRDR2 at 3.4 Angstroms resolution	5.33	3.4	2021-12-07	2022-06-08
31913	7vdc	3.28 A structure of the rabbit muscle aldolase	0.0075	3.28	2021-09-06	2021-12-29
15028	7zyy	Cryo-EM structure of Lactococcus lactis pyruvate carboxylase with acetyl-CoA	0.187	2.12	2022-05-25	2022-10-12
22836	7kea	SARS-CoV-2 D614G 1-RBD-up Spike Protein Trimer without the P986-P987 stabilizing mutations (S-GSAS-D614G sub classification)	0.2	3.33	2020-10-10	2020-11-04
13179	7p3f	Streptomyces coelicolor dATP/ATP-loaded NrdR in complex with its cognate DNA	0.4	3.31	2021-07-07	2022-05-11
26055	7tph	Delta (B.1.617.2) SARS-CoV-2 variant spike protein (S-GSAS-Delta) in the 2-RBD-up conformation - D3	0.8	3.58	2022-01-25	2022-02-09
28269	8en7	Mouse apoferritin heavy chain without zinc determined using single-particle cryo-EM with Apollo camera.	0.06	1.68	2022-09-28	2022-12-21
22755	7k9x	Aldolase, rabbit muscle (beam-tilt refinement x1)	0.022	3.8	2020-09-29	2020-12-02
31489	7f8j	Cryo-EM structure of human pannexin-1 in a nanodisc	0.01	3.6	2021-07-02	2022-01-26

11676	7a7a	Cryo-EM structure of W107R after heme uptake (2heme molecules) KatG from M. tuberculosis	0.107	3.08	2020-08-27	2021-01-27
0510	6nts	Protein Phosphatase 2A (Aalpha-B56alpha-Calpha) holoenzyme in complex with a Small Molecule Activator of PP2A (SMAP)	0.3	3.63	2019-01-30	2020-05-06
26361	7u66	Structure of E. coli dGTPase bound to T7 bacteriophage protein Gp1.2 and dGTP	0.199	3.1	2022-03-03	2022-08-31
31712	7v4i	Cryo-EM Structure of Camellia sinensis glutamine synthetase CsGSIb decamer assembly	0.018	3.3	2021-08-13	2022-05-18
0751	6kq8	328 K cryoEM structure of Sso-KARI in complex with Mg2+	1.42	3.0	2019-08-16	2020-03-25
16788	8cpw	Human apoferritin after 405 nm + 488 nm laser exposure in presence of rsEGFP2	0.025	1.79	2023-03-03	2023-03-15
28274	8enn	Homocitrate-deficient nitrogenase MoFe-protein from Azotobacter vinelandii nifV knockout	0.288	2.58	2022-09-30	2023-03-08
30988	7e4x	Structure of Enolase from Mycobacterium tuberculosis	0.0467	3.08	2021-02-15	2022-02-16
23312	7lg6	BG505 SOSIP.v5.2 in complex with VRC40.01 and RM19R Fabs	0.46	3.28	2021-01-19	2021-09-15
23947	7mqh	Bartonella henselae NrnC complexed with pAAAGG in the presence of Ca2+. D4 Symmetry.	0.0697	3.1	2021-05-05	2021-09-15
8946	6e0f	Mitochondrial peroxiredoxin from Leishmania infantum in complex with unfolding client protein after heat stress	0.0341	3.7	2018-07-06	2019-02-20
24530	7rlh	Cryo-EM structure of human p97-D592N mutant bound to ATPgS.	0.25	3.0	2021-07-23	2021-09-22
32744	7ws5	Structures of Omicron Spike complexes illuminate broad-spectrum neutralizing antibody development	0.2036	3.7	2022-01-28	2022-06-01
24526	7rld	Cryo-EM structure of human p97-E470D mutant bound to ADP.	0.15	3.4	2021-07-23	2021-09-22
22337	7jhh	Cryo-EM structure of ATP-bound fully inactive AMPK in complex with Fab and nanobody	0.015	3.92	2020-07-20	2021-07-21
9116	6mhq	CryoEM reconstruction of native lens connexin-46/50 at 3.4 angstrom resolution	0.03	3.4	2018-09-18	2018-12-12
25985	7tla	Down-state locked rS2d SARS-CoV-2 spike ectodomain in the RBD-down conformation, State 1	0.489	3.13	2022-01-18	2022-02-02
21459	6vyg	Cryo-EM structure of Plasmodium vivax hexokinase (Closed state)	0.014	3.5	2020-02-26	2020-05-06
11151	6zbd	Merozoite surface protein 1 (MSP-1) from Plasmodium falciparum, alternative conformation 2	0.266	3.21	2020-06-08	2021-05-19
13819	7q4w	CryoEM structure of electron bifurcating Fe-Fe hydrogenase HydABC complex A. woodii in the oxidised state	0.0642	3.78	2021-11-02	2023-02-15
32509	7whr	Cryo-EM Structure of Leishmanial GDP-mannose pyrophosphorylase	0.072	3.4	2021-12-31	2022-09-21
21575	6w8n	structure of a trans-membrane protein	0.453	3.2	2020-03-20	2021-08-04
20706	6ua5	Human IMPDH2 treated with ATP, IMP, NAD+, and 2 mM GTP. Free interfacial octamer reconstruction.	0.1	3.79	2019-09-10	2020-03-25
27080	8cyj	RBD of SARS-CoV-2 Spike protein in complex with pan-sarbecovirus nanobodies 2-10, 2-67, 2-62 and 1-25	0.1	3.6	2022-05-23	2022-07-06
21708	6wkt	Cu(I)-bound Copper Storage Protein BsCsp3	4	3.4	2020-04-16	2021-04-21
23708	7m74	ATP-bound AMP-activated protein kinase	0.009	3.93	2021-03-26	2021-12-15
30499	7cyf	Cryo-EM structure of bicarbonate transporter SbtA in complex with PII-like signaling protein SbtB from Synechocystis sp. PCC 6803	0.03	3.15	2020-09-03	2021-06-23
0749	6kpk	309 K cryoEM structure of Sso-KARI in complex with Mg2+	1.43	2.3	2019-08-15	2020-03-25
11330	6zoy	Structure of Disulphide-stabilized SARS-CoV-2 Spike Protein Trimer (x1 disulphide-bond mutant, S383C, D985C, K986P, V987P, single Arg S1/S2 cleavage site) in Closed State	0.0211	3.1	2020-07-08	2020-07-22
20691	6u8s	Human IMPDH2 treated with ATP, IMP, NAD+, and 2 mM GTP. Filament assembly interface reconstruction.	17	3.14	2019-09-05	2020-03-25

25225	7sng	structure of G6PD-WT tetramer	0.4	2.8	2021-10-28	2022-07-13
13182	7p3q	Streptomyces coelicolor dATP/ATP-loaded NrdR octamer	0.4	3.12	2021-07-08	2022-05-11
14435	7z0z	Abortive infection DNA polymerase AbiK from Lactococcus lactis, Y44F variant	0.5	2.68	2022-02-24	2022-09-07
11852	7apd	Bovine Papillomavirus E1 DNA helicase-replication fork complex	3	3.9	2020-10-16	2021-11-17
20224	6ozc	BG505 SOSIP.664 with 2G12 Fab2	0.3	3.79	2019-05-15	2020-05-20
31915	7vde	3.6 A structure of the human hemoglobin	0.0004	3.6	2021-09-06	2021-12-29
8908	6drv	Cryo-EM structure of beta-galactosidase using RELION on Amazon Web Services	0.0233	2.2	2018-06-13	2018-07-11
21811	6wkw	EM structure of CtBP2 with a minimal dehydrogenase domain of CtBP2	0.042	3.6	2020-04-17	2020-12-02
10903	6ysn	Human TRPC5 in complex with Pico145 (HC-608)	0.045	3.0	2020-04-22	2020-12-02
20041	6of3	Precursor ribosomal RNA processing complex, State 1.	4.5	3.0	2019-03-28	2019-09-11
9391	6njp	Structure of the assembled ATPase EscN in complex with its central stalk EscO from the enteropathogenic E. coli (EPEC) type III secretion system	0.05	3.29	2019-01-03	2019-02-20
22168	6xfa	Cryo-EM structure of EBV BFLF1	0.0281	3.6	2020-06-15	2021-02-24
30601	7d74	Structure of a protein	0.0365	3.1	2020-10-02	2021-05-19
27796	8dzf	Cryo-EM structure of bundle-forming pilus extension ATPase from E.coli in the presence of AMP-PNP (class-2)	0.15	3.69	2022-08-07	2022-10-26
21257	6vo1	BG505 SOSIP.v5.2 in complex with rhesus macaque Fab RM20J	0.43	3.88	2020-01-29	2020-07-01
20742	6udp	Human IMPDH2 treated with ATP, IMP, and 20 mM GTP. Filament assembly interface reconstruction.	0.02	2.95	2019-09-19	2020-03-25
30766	7dmp	Mouse radial spoke complex	0.018	3.2	2020-12-05	2021-07-21
0721	6klx	Pore structure of Iota toxin binding component (Ib)	0.035	2.9	2019-07-30	2020-01-15
15212	8a6t	Cryo-EM structure of the electron bifurcating Fe-Fe hydrogenase HydABC complex from Thermoanaerobacter kivui in the reduced state	0.0618	3.1	2022-06-19	2023-02-15
14587	7zbn	Cryo-EM structure of the human GS-GN complex in the inhibited state	0.025	2.62	2022-03-23	2022-06-22
27174	8d44	Cryo-electron microscopy structure of human kidney Fructose-bisphosphate aldolase B	0.15	2.8	2022-06-01	2022-11-16
20687	6u8e	Human IMPDH2 treated with ATP, IMP, and NAD+. Filament assembly interface reconstruction.	0.1	3.03	2019-09-04	2020-03-25
23325	7lgj	Cyanophycin synthetase 1 from Synechocystis sp. UTEX2470 with ADPCP and 8x(Asp-Arg)-NH2	0.68	2.6	2021-01-20	2021-08-18
31562	7fes	Cryo-EM structure of apo BsClpP at pH 4.2	0.6	3.4	2021-07-21	2022-07-06
9104	6mdr	Cryo-EM structure of the Ceru+32/GFP-17 protomer	0.05	3.47	2018-09-05	2019-01-23
21917	6wun	Mitochondrial SAM complex - dimer 3 in detergent	0.6	3.9	2020-05-04	2020-08-12
10692	6y5a	Serotonin-bound 5-HT3A receptor in Salipro	0.1	2.8	2020-02-25	2020-12-23
24528	7rlf	Cryo-EM structure of human p97-E470D mutant bound to ATPgS.	0.1	3.1	2021-07-23	2021-09-22
33315	7xnh	Human Cx36/GJD2 gap junction channel with pore-lining N-terminal helices in soybean lipids	0.01	3.1	2022-04-28	2023-03-22
14774	7zl1	PTX3 Pentraxin Domain	0.0169	2.5	2022-04-13	2022-08-03
26733	7usd	Cryo-EM structure of D-site Rac1-bound WAVE Regulatory Complex	0.023	3.0	2022-04-25	2022-09-21
26961	8csa	Triple mutant (K417N-E484K-N501Y) SARS-CoV-2 spike protein in the 3-RBD-Down conformation (S-GSAS-D614G-K417N-E484K-N501Y)	0.2	3.84	2022-05-12	2022-07-20
20994	6v0d	Lipophilic Envelope-spanning Tunnel B (LetB), Map 2	0.042	3.49	2019-11-18	2020-05-06
13474	7pko	CryoEM structure of Rotavirus NSP2	0.0283	3.9	2021-08-26	2021-09-29
27699	8dti	Cryo-EM structure of Arabidopsis SPY in complex with GDP-fucose	5.4	3.8	2022-07-25	2023-03-08

26479	7ufs	Cryo-EM Structure of Bl_Man38B at 3.4 A	3.5	3.4	2022-03-23	2022-11-16
3999	6ezj	Imidazoleglycerol-phosphate dehydratase	0.158	3.1	2017-11-15	2018-02-07
13316	7pc2	HIV-1 Env (BG505 SOSIP.664) in complex with the IgA bNAb 7-269 and the antibody 3BNC117.	0.29	2.8	2021-08-03	2022-02-23
4907	6rkd	Molybdenum storage protein under turnover conditions	0.068	3.2	2019-04-30	2019-12-18
12696	7o1q	Amyloid beta oligomer displayed on the alpha hemolysin scaffold	0.006	3.4	2021-03-30	2021-04-14
22521	7jy5	Map of human p97 in complex with ATPgammaS and Npl4/Ufd1 (masked around p97 and C6 averaged)	0.018	2.89	2020-08-28	2021-01-20
28275	8eno	Homocitrate-deficient nitrogenase MoFe-protein from A. vinelandii nifV knockout in complex with NafT	0.125	2.71	2022-09-30	2023-03-08
16784	8cps	Human apoferritin	0.025	1.82	2023-03-03	2023-03-15
30498	7cye	Cryo-EM structure of sodium-dependent bicarbonate transporter SbtA from Synechocystis sp. PCC 6803	0.025	3.54	2020-09-03	2021-06-23
13581	7ppj	human SLFN5	0.707	3.44	2021-09-14	2022-01-26
20642	6u59	HIV-1 B41 SOSIP.664 in complex with rabbit antibody 13B	0.53	3.86	2019-08-27	2020-01-29
28259	8emq	Mouse apoferritin heavy chain with zinc determined using single-particle cryo-EM with Apollo camera.	0.06	1.66	2022-09-28	2022-12-21
25227	7sni	Structure of G6PD-D200N tetramer bound to NADP+ and G6P	0.42	2.5	2021-10-28	2022-07-13
13691	7px8	CryoEM structure of mammalian acylaminoacyl-peptidase	0.012	3.27	2021-10-08	2022-05-25
32540	7wiy	Cryo-EM structure of human TPH2 tetramer	0.4	3.09	2022-01-05	2022-10-05
4754	6r8b	Escherichia coli AGPase in complex with FBP.	0.5	3.1	2019-04-01	2020-02-05
15389	8aey	3 A CRYO-EM STRUCTURE OF MYCOBACTERIUM TUBERCULOSIS FERRITIN FROM TIMEPIX3 detector	6.5	3.05	2022-07-14	2023-01-18
0485	6nqd	Cryo-EM structure of T/F100 SOSIP.664 HIV-1 Env trimer in complex with 8ANC195 Fab	0.01	3.9	2019-01-21	2019-03-06
24981	7sbk	Closed state of pre-fusion SARS-CoV-2 Delta variant spike protein	0.1	3.1	2021-09-25	2021-11-03
27175	8d45	Cryo-EM structure of human Kidney Betaine-Homocysteine Methyltransferase	0.15	2.62	2022-06-01	2022-11-16
24540	7rlt	Structure of ligand-free ALDH1L1 (10-formyltetrahydrofolate dehydrogenase)	0.04	3.7	2021-07-26	2022-01-12
12740	7o6q	Structure of the borneol dehydrogenase 1 of salvia rosmarinus	0.3	1.88	2021-04-12	2021-12-01
22758	7ka4	Aldolase, rabbit muscle (beam-tilt refinement x4)	0.0201	2.8	2020-09-29	2020-12-02
26161	7txv	Cyanophycin synthetase 1 from Synechocystis sp. UTEX2470 E82Q with ATP and 16x(Asp-Arg)	0.59	2.7	2022-02-09	2022-06-01
26442	7uc2	Structure of G6PD-D200N tetramer bound to NADP+ with no symmetry applied	0.4	2.5	2022-03-15	2022-09-14
12591	7ntm	Cryo-EM structure of S.cerevisiae native alcohol dehydrogenase 1 (ADH1) in its tetrameric apo state	0.185	2.86	2021-03-10	2022-10-12
22837	7keb	SARS-CoV-2 D614G 1RBD up Spike Protein Trimer without the P986-P987 stabilizing mutations (S-GSAS-D614G sub-classification)	0.2	3.48	2020-10-10	2020-11-04
11155	6zbh	Merozoite surface protein 1 (MSP-1) from Plasmodium falciparum, alternative conformation 5	0.3	3.6	2020-06-08	2021-05-19
0690	6j6k	The reconstruction of apo-state streptavidin at 3.3 Angstrom resolution	0.084	3.3	2019-01-15	2019-05-29
23893	7mke	Cryo-EM structure of Escherichia coli RNA polymerase bound to lambda PR promoter DNA (class 2)	0.19	3.7	2021-04-23	2021-09-29
13108	7oy2	High resolution structure of cytochrome bd-II oxidase from E. coli	0.012	2.06	2021-06-23	2021-12-15
26831	7uwf	Human Rix1 sub-complex scaffold	1.04	2.7	2022-05-03	2022-11-02
13753	7q13	Human GYS1-GYG1 complex activated state bound to glucose-6-phosphate, uridine diphosphate, and glucose	0.03	3.0	2021-10-17	2022-07-27

26360	7u65	Structure of E. coli dGTPase bound to T7 bacteriophage protein Gp1.2	0.171	2.8	2022-03-03	2022-08-31
22352	7jji	Structure of SARS-CoV-2 3Q-2P full-length prefusion spike trimer (C3 symmetry)	0.25	3.6	2020-07-26	2020-08-26
9913	6k43	Cryo-EM structure of Holo-bacterioferritin-form-I from Streptomyces coelicolor	5.1	3.7	2019-05-23	2021-02-03
16789	8cpx	Human apoferritin after 488 nm laser exposure in presence of rsEGFP2	0.025	1.76	2023-03-03	2023-03-15
23048	7kve	Cryo-EM structure of human Factor V at 3.3 Angstrom resolution	0.43	3.3	2020-11-27	2021-03-10
13050	7osg	ABC Transporter complex NosDFYL, consensus refinement	0.02	3.3	2021-06-08	2022-06-22
13665	7pv4	PhiCPV4 bacteriophage Portal Protein	0.023	2.8	2021-10-01	2022-10-12
11211	6zh2	Cryo-EM structure of DNA-PKcs (State 1)	0.055	3.92	2020-06-20	2020-10-21
10495	6tg9	Cryo-EM Structure of NADH reduced form of NAD+-dependent Formate Dehydrogenase from Rhodobacter capsulatus	0.0223	3.24	2019-11-15	2020-04-22
13320	7pch	Human carboxyhemoglobin bound to Staphylococcus aureus hemophore IsdB - 1:2 complex	0.04	2.89	2021-08-03	2022-04-13
0731	6kng	CryoEM map and model of Nitrite Reductase at pH 8.1	0.0405	2.85	2019-08-05	2020-08-12
13364	7pf1	UVC treated Human apoferritin	0.0125	2.1	2021-08-11	2022-01-26
28147	8ehg	Rabbit muscle aldolase determined using single-particle cryo-EM with Apollo camera.	1.23	2.24	2022-09-14	2022-12-21
30600	7d73	Cryo-EM structure of GMPPA/GMPPB complex bound to GTP (State I)	0.0389	3.0	2020-10-02	2021-05-05
12738	7o6e	2.12 A cryo-EM structure of Mycobacterium tuberculosis Ferritin	7	2.1	2021-04-10	2021-05-19
26362	7u67	Structure of E. coli dGTPase bound to T7 bacteriophage protein Gp1.2 and GTP	0.193	2.5	2022-03-03	2022-08-31
13469	7pke	C-reactive protein pentamer at pH 7.5 with phosphocholine ligand	0.0167	3.3	2021-08-25	2021-12-22
21371	6vrs	Single particle reconstruction of glucose isomerase from Streptomyces rubiginosus based on data acquired in the presence of substantial aberrations	5	2.7	2020-02-09	2020-02-19
21408	6vvz	Mycobacterium tuberculosis RNAP S456L mutant transcription initiation intermediate structure with Sorangicin	0.394	3.72	2020-02-18	2020-10-21
21376	6vsc	Single particle reconstruction of HemQ from Geobacillus based on data acquired in the presence of substantial aberrations	0.75	2.6	2020-02-11	2020-02-19
20707	6uaj	Human IMPDH2 treated with ATP, IMP, NAD+, and 2 mM GTP. Free canonical octamer reconstruction.	0.1	3.84	2019-09-10	2020-03-25
14385	7yyo	Cryo-EM structure of an a-carboxysome RuBisCO enzyme at 2.9 A resolution	1.18	2.87	2022-02-18	2023-01-25
9041	6eec	Mycobacterium tuberculosis RNAP promoter unwinding intermediate complex with RbpA/CarD and AP3 promoter captured by Corallopyronin	0.3	3.55	2018-08-13	2018-11-21
26397	7u9g	Rabies virus glycoprotein pre-fusion trimer in complex with neutralizing antibody RVA122	0.007	3.39	2022-03-10	2022-07-20
21652	6wfq	NanR dimer-DNA hetero-complex	0.01	3.9	2020-04-03	2021-03-10
13178	7p37	Streptomyces coelicolor ATP-loaded NrdR	0.35	2.96	2021-07-07	2022-05-11
12938	7oj5	Cryo-EM structure of Medicago truncatula HISN5 protein	0.076	2.4	2021-05-13	2021-06-02
25570	7szj	Cryo-EM structure of Rifamycin bound to E. coli RNAP and rrnBP1 promoter complex	0.4	3.11	2021-11-28	2022-07-13
31497	7f94	Structure of C-terminal truncated connexin43/Cx43/GJA1 gap junction intercellular channel with two conformationally different hemichannels	0.008	3.6	2021-07-03	2022-07-06

20521	6pxm	Horse spleen apoferritin light chain	0.8	2.1	2019-07-26	2019-08-07
10101	6s61	Apoferritin from mouse at 1.84 angstrom resolution	0.04	1.84	2019-07-02	2019-07-10
23608	7lzj	DpK2 bacteriophage tail spike depolymerase	0.035	2.7	2021-03-10	2021-08-25
33861	7yim	Cryo-EM structure of human Alpha-fetoprotein	0.41	2.6	2022-07-17	2023-01-18
12043	7b5p	AcrB in cycloalkane amphipol	0.0224	3.2	2020-12-05	2021-12-08
0747	6kpi	298 K cryoEM structure of Sso-KARI in complex with Mg2+	1.55	2.43	2019-08-15	2020-03-25
11336	6zp5	SARS-CoV-2 spike in prefusion state (flexibility analysis, 1-up closed conformation)	0.2	3.1	2020-07-08	2020-07-29
20217	6owt	Structure of SIVsmm Nef and SMM tetherin bound to the clathrin adaptor AP-2 complex	0.485	3.8	2019-05-10	2019-09-25
21458	6vyf	Cryo-EM structure of Plasmodium vivax hexokinase (Open state)	0.012	3.3	2020-02-26	2020-05-06
15528	8amx	AQP7 dimer of tetramers_D4	0.2	2.55	2022-08-04	2023-02-15
0988	6lvb	Structure of Dimethylformamidase, tetramer	0.07	2.8	2020-02-02	2020-06-03
23807	7mey	Structure of yeast Ubr1 in complex with Ubc2 and monoubiquitinated N-degron	0.015	3.67	2021-04-08	2021-11-24
13960	7qh2	Cryo-EM structure of Ldh-EtfAB complex from Acetobacterium woodii	0.0102	2.43	2021-12-10	2022-06-29
0488	6nr3	Cryo-EM structure of the TRPM8 ion channel in complex with high occupancy icilin, PI(4,5)P2, and calcium	0.025	3.4	2019-01-22	2019-02-20
24629	7rq7	CLC-ec1 at pH 4.5 100mM Cl TWIST2	0.171	3.95	2021-08-05	2022-11-16
22807	7kcb	The Cryo-EM Structure of Alcohol Dehyrogenase from Yeast in complex with NAD+ and Trifluoro Ethanol (TFE)	1.5	2.77	2020-10-05	2021-03-31
10199	6shj	Escherichia coli AGPase in complex with FBP. Symmetry applied C2	0.18	3.2	2019-08-07	2020-02-05
25224	7snf	Structure of G6PD-WT dimer	0.28	3.5	2021-10-28	2022-07-13
0689	6j6j	The reconstruction of biotin-bound streptavidin at 3.2 Angstrom resolution	0.12	3.2	2019-01-15	2019-05-29
9798	6jcv	Cryo-EM structure of Sulfolobus solfataricus ketol-acid reductoisomerase (Sso-KARI) with Mg2+ at pH7.5	2.6	2.92	2019-01-30	2019-04-17
20812	6ukt	Cryo-EM structure of mammalian Ric-8A:Galpha(i):nanobody complex	4.07	3.87	2019-10-05	2020-03-11
11156	6zbj	Plasmodium falciparum merozoite surface protein 1 dimer, conformation 1	0.247	3.3	2020-06-08	2021-05-19
16786	8cpu	Human apoferritin after 561 nm laser exposure	0.025	1.76	2023-03-03	2023-03-15
21576	6w8o	Structure of an Apo membrane protein	0.431	3.4	2020-03-20	2021-08-04
0748	6kpj	298 K cryoEM structure of Sso-KARI in complex with Mg2+, NADH and CPD	1.42	2.56	2019-08-15	2020-03-25
10201	6shn	Escherichia coli AGPase in complex with FBP. Symmetry C1	0.13	3.3	2019-08-07	2020-02-05
22682	7k5k	Plasmodium vivax M17 leucyl aminopeptidase Pv-M17	0.4	2.66	2020-09-17	2020-12-16
0989	6lvc	Structure of Dimethylformamidase, dimer	0.066	3.0	2020-02-02	2020-06-03
30785	7dnj	K63-polyUb MDA5CARDs complex	0.513	3.3	2020-12-09	2021-10-13
21375	6vsb	Prefusion 2019-nCoV spike glycoprotein with a single receptor-binding domain up	0.19	3.46	2020-02-10	2020-02-26
23210	7l7b	Clostridioides difficile RNAP with fidaxomicin	0.4	3.26	2020-12-28	2022-02-02
24529	7rlg	Cryo-EM structure of human p97-D592N mutant bound to ADP.	0.15	3.7	2021-07-23	2021-09-22
23311	7lg5	Synechocystis sp. UTEX2470 Cyanophycin synthetase 1 with ATP	0.22	2.63	2021-01-19	2021-08-18
30583	7d5k	CryoEM structure of cotton cellulose synthase isoform 7	0.2	3.5	2020-09-26	2021-07-28
13062	7otm	Cryo-EM structure of DNA-PKcs in complex with NU7441	0.2	3.33	2021-06-10	2022-01-12
32083	7vq2	Structure of Apo-hsTRPM2 channel TM domain	0.0022	3.68	2021-10-18	2021-12-22
27010	8cvn	CRYO-EM STRUCTURE OF HUMAN 15-PGDH IN COMPLEX WITH SMALL MOLECULE SW209415	0.08	2.4	2022-05-18	2023-03-01
9799	6jcw	Cryo-EM Structure of Sulfolobus solfataricus ketol-acid reductoisomerase (Sso-KARI) with Mg2+ at pH8.5	1.88	3.04	2019-01-30	2019-04-17

13476	7pkp	NSP2 RNP complex	0.0205	3.1	2021-08-26	2021-09-29
9390	6njo	Structure of the assembled ATPase EscN from the enteropathogenic E. coli (EPEC) type III secretion system	0.05	3.34	2019-01-03	2019-02-20
21442	6vxk	Cryo-EM Structure of the full-length A39R/PlexinC1 complex	0.009	3.1	2020-02-22	2020-04-29
7006	6aui	Human ribonucleotide reductase large subunit (alpha) with dATP and CDP	0.0275	3.3	2017-09-01	2018-04-18
10691	6y59	5-HT3A receptor in Salipro (apo, C5 symmetric)	0.1	3.2	2020-02-25	2020-12-23
26428	7ual	Structure of G6PD-D200N tetramer bound to NADP+ and G6P with no symmetry applied	0.2	2.9	2022-03-13	2022-09-14
13641	7ptv	Structure of the Mimivirus genomic fibre asymmetric unit	0.028	3.3	2021-09-27	2022-08-10
24852	7s67	Extended conformation of daytime state KaiC	0.007	3.8	2021-09-13	2021-09-22
32211	7vyq	Short chain dehydrogenase (SCR) cryoEM structure with NADP and ethyl 4-chloroacetoacetate	0.3	3.13	2021-11-15	2022-04-06
10208	6si8	Escherichia coli AGPase in complex with AMP.	0.25	3.4	2019-08-09	2020-02-05
30556	7d3f	Cryo-EM structure of human DUOX1-DUOXA1 in high-calcium state	0.025	2.6	2020-09-19	2020-12-09
27892	8e4m	The intermediate C2-state mouse TRPM8 structure in complex with the cooling agonist C3 and PI(4,5)P2	0.14	3.44	2022-08-18	2022-10-26
21997	6x29	SARS-CoV-2 rS2d Down State Spike Protein Trimer	0.383	2.7	2020-05-20	2020-05-27
24547	7rlu	Structure of ALDH1L1 (10-formyltetrahydrofolate dehydrogenase) in complex with NADP	0.02	2.9	2021-07-26	2022-01-12
31968	7vgg	Cryo-EM structure of Ultraviolet-B activated UVR8 in complex with COP1	0.35	3.1	2021-09-16	2022-05-04
9971	6kff	Undocked INX-6 hemichannel in a nanodisc	0.03	3.8	2019-07-07	2020-02-12
22824	7kdj	SARS-CoV-2 D614G 1-RBD-up Spike Protein Trimer fully cleaved by furin without the P986-P987 stabilizing mutations (S-RRAR-D614G)	0.16	3.49	2020-10-08	2020-11-04
4905	6rjh	3D structure of horse spleen apoferritin determined using multifunctional graphene supports for electron cryomicroscopy	0.04	2.1	2019-04-26	2019-06-05
0636	6o6r	Structure of the TRPM8 cold receptor by single particle electron cryo-microscopy, AMTB-bound state	0.035	3.2	2019-03-07	2019-09-18
11154	6zbg	Merozoite surface protein 1 (MSP-1) from Plasmodium falciparum, alternative conformation 4	0.3	3.2	2020-06-08	2021-05-19
13159	7p1h	Structure of the V. vulnificus ExoY-G-actin-profilin complex	0.0025	3.9	2021-07-01	2021-11-17
26458	7uds	Structure of lineage I (Pinneo) Lassa virus glycoprotein bound to Fab 25.10C	0.03	3.1	2022-03-20	2022-06-15
22305	6xss	CryoEM structure of designed helical fusion protein C4_nat_HFuse-7900	0.0158	3.7	2020-07-16	2020-12-23
10693	6y5b	5-HT3A receptor in Salipro (apo, asymmetric)	0.1	3.1	2020-02-25	2020-12-23
26030	7toe	Structure of G6PD-WT tetramer with no symmetry imposed	0.3	3.0	2022-01-24	2022-09-14
0742	6kpa	277 K cryoEM structure of Sso-KARI in complex with Mg2+, NADH and CPD	1.5	2.75	2019-08-15	2020-03-25
23411	7ll1	Cryo-EM map of BG505 DS-SOSIP in complex with glycan276-dependent broadly neutralizing antibody VRC40.01 Fab	1.35	3.73	2021-02-03	2021-03-17
16785	8cpt	Human apoferritin after 488 nm laser exposure	0.025	1.79	2023-03-03	2023-03-15
23012	7krs	Structural impact on SARS-CoV-2 spike protein by D614G substitution	0.005	3.2	2020-11-20	2021-03-24
23187	7l5j	Mouse Norovirus Protruding domain complexed with neutralizing Fab fragment from mAb A6.2	0.31	3.2	2020-12-22	2021-04-07
24984	7sbp	Closed state of pre-fusion SARS-CoV-2 Kappa variant spike protein	0.1	3.1	2021-09-25	2021-11-03
9910	6k3o	Cryo-EM structure of Apo-bacterioferritin from Streptomyces coelicolor	4.95	3.4	2019-05-21	2021-02-03
22902	7kjy	The Cryo-EM Structure of Alcohol Dehyrogenase from Yeast in complex with NADH - Open Form	0.019	3.2	2020-10-26	2021-03-31

13499	7pls	Cryo-EM structures of human fucosidase FucA1 reveal insight into substate recognition and catalysis.	0.03	2.49	2021-09-01	2022-08-10
26667	7upi	Cryo-EM structure of SHOC2-PP1c-MRAS holophosphatase complex	0.012	2.89	2022-04-15	2022-05-04
7113	6bly	Cryo-EM structure of human CPSF-160-WDR33 complex at 3.36 A resolution	0.06	3.36	2017-11-12	2017-11-22
9592	6ach	Structure of NAD+-bound leucine dehydrogenase from Geobacillus stearothermophilus by cryo-EM	0.05	3.2	2018-07-26	2018-12-26
26478	7ufr	Cryo-EM Structure of Bl_Man38A at 2.7 A	5	2.7	2022-03-23	2022-11-16
24722	7rx2	afTMEM16 in C22 lipid nanodiscs with MSP1E3 scaffold protein in the presnece of Ca2+	0.013	2.7	2021-08-21	2022-05-18
4668	6qxf	Cas1-Cas2-Csn2-DNA complex from the Type II-A CRISPR-Cas system	0.03	3.6	2019-03-07	2019-05-08
10496	6tga	Cryo-EM Structure of as isolated form of NAD+-dependent Formate Dehydrogenase from Rhodobacter capsulatus	0.0112	3.26	2019-11-15	2020-04-22
6830	5yi5	human ferritin mutant - E-helix deletion	0.3	3.0	2017-10-02	2018-02-21
32756	7wsf	Cryo-EM structure of SARS-CoV spike receptor-binding domain in complex with minke whale ACE2	0.23	2.87	2022-01-29	2022-10-19
32510	7whs	Cryo-EM Structure of Leishmanial GDP-mannose pyrophosphorylase in complex with GTP	0.199	3.1	2021-12-31	2022-09-21
33600	7y42	Cryo-EM structure of the SARS-CoV-2 spike glycoprotein in complex with all-trans retinoic acid	0.175	3.45	2022-06-13	2022-07-06
22610	7k0r	Nucleotide bound SARS-CoV-2 Nsp15	1.1	3.3	2020-09-04	2020-12-09
21904	6wu3	Structure of VcINDY-Na+ in amphipol	7.01	3.16	2020-05-04	2020-09-16
33309	7xn3	E.coli phosphoribosylpyrophosphate (PRPP) synthetase type B filament bound with Pi	0.035	2.9	2022-04-27	2022-06-29
11157	6zbl	Plasmodium falciparum merozoite surface protein 1 dimer, conformation 2	0.24	3.6	2020-06-08	2021-05-19
20080	6ois	CryoEM structure of Arabidopsis DR complex (DMS3-RDM1)	0.05	3.6	2019-04-09	2019-07-24
13520	7pm4	Cryo-EM structures of human fucosidase FucA1 reveal insight into substate recognition and catalysis.	0.018	2.49	2021-09-01	2022-08-10
10337	6sxa	XPF-ERCC1 Cryo-EM Structure, Apo-form	0.3	3.6	2019-09-25	2020-03-11
30083	6m52	Human apo ferritin chain A frozen on TEM grid with amorphous carbon supporting film	0.0457	2.6	2020-03-09	2020-05-13
11587	6zzu	Partial structure of the substrate-free tyrosine hydroxylase (apo-TH).	0.6	3.5	2020-08-05	2021-11-17
22156	6xey	Cryo-EM structure of the SARS-CoV-2 spike glycoprotein bound to Fab 2-4	0.22	3.25	2020-06-14	2020-07-22
20708	6uan	B-Raf:14-3-3 complex	0.014	3.9	2019-09-11	2019-09-25
7114	6bm0	Cryo-EM structure of human CPSF-160-WDR33 complex at 3.8 A resolution	0.06	3.8	2017-11-12	2017-11-22
12806	7ocf	Active state GluA1/A2 AMPA receptor in complex with TARP gamma 8 and CNIH2 (LBD-TMD)	0.02	3.6	2021-04-26	2021-06-09
31946	7vf2	Human m6A-METTL associated complex (WTAP, VIRMA, ZC3H13, and HAKAI)	0.36	3.0	2021-09-10	2022-09-14
30535	7d0i	Cryo-EM structure of Schizosaccharomyces pombe Atg9	0.0161	3.0	2020-09-10	2020-10-28
24121	7n1q	Structural basis for enhanced infectivity and immune evasion of SARS-CoV-2 variants	0.25	2.9	2021-05-28	2021-07-07
0499	6nsk	CryoEM structure of Helicobacter pylori urea channel in open state.	0.06	2.7	2019-01-24	2019-04-03
27945	8e8o	Cryo-EM structure of human ME3 in the presence of citrate	0.02	2.77	2022-08-25	2023-02-08
10370	6t2v	Cryo-EM structure of the RecBCD in complex with Chi-plus2 substrate	0.02	3.8	2019-10-09	2020-01-01
32960	7x27	MERS-CoV spike complex	0.005	2.49	2022-02-25	2023-03-22
26354	7u5l	Cryo-EM Structure of Ferritin	0.2	2.67	2022-03-02	2022-12-14
20042	6of4	Precursor ribosomal RNA processing complex, apo-state.	4.5	3.2	2019-03-28	2019-09-11

25804	7tc7	Cryo-EM structure of methane monooxygenase hydroxylase (by quantifoil)	0.8	2.9	2021-12-23	2023-01-25
7436	6c9i	Single-Particle reconstruction of DARP14 - A designed protein scaffold displaying ~17kDa DARPin proteins - Scaffold	0.0164	3.09	2018-01-26	2018-03-21
27670	8dr4	Open state of RFC:PCNA bound to a 3' ss/dsDNA junction (DNA2) without NTD	0.2	2.45	2022-07-20	2022-08-17
31104	7eg1	Cryo-EM structure of DNMDP-induced PDE3A-SLFN12 complex	0.0188	3.2	2021-03-23	2021-11-03
0631	6o6a	Structure of the TRPM8 cold receptor by single particle electron cryo-microscopy, ligand-free state	0.02	3.6	2019-03-05	2019-09-18
4761	6r8u	Escherichia coli AGPase in complex with AMP.	0.31	3.0	2019-04-02	2020-02-05
22279	6xot	CryoEM structure of human presequence protease in partial open state 2	0.005	3.9	2020-07-07	2021-07-07
23328	7lgq	Cyanophycin synthetase 1 from Synechocystis sp. UTEX2470 with ATP and 8x(Asp-Arg)-Asn	0.65	2.7	2021-01-20	2021-08-18
22910	7kkl	SARS-CoV-2 Spike bound to mNb6 in closed conformation	2	2.85	2020-10-27	2020-11-11
15961	8bc3	Cryo-EM Structure of a BmSF-TAL - Sulfofructose Schiff Base Complex	0.0259	2.1	2022-10-14	2023-01-18
22416	7jpn	Cryo-EM structure of Arpin-bound Arp2/3 complex	0.154	3.24	2020-08-09	2022-02-09
9196	6mrd	ADP-bound human mitochondrial Hsp60-Hsp10 half-football complex	0.525	3.82	2018-10-12	2020-04-15
21959	6wxh	Colicin E1 fragment in nanodisc-embedded TolC	0.15	3.09	2020-05-10	2021-05-12
13054	7osl	Cryo-EM structure of nonameric EPEC SctV-C	0.0234	3.1	2021-06-09	2021-09-29
30599	7d72	Cryo-EM structures of human GMPPA/GMPPB complex bound to GDP-Mannose	0.02	3.4	2020-10-02	2021-05-05
33540	7y0d	Cryo-EM structure of the Mycobacterium smegmatis DNA integrity scanning protein (MsDisA).	1.4	3.1	2022-06-04	2023-02-08
0752	6kqj	309 K cryoEM structure of Sso-KARI in complex with Mg2+, NADH and CPD	1.55	2.54	2019-08-18	2020-03-25
31560	7feq	Cryo-EM structure of apo BsClpP at pH 6.5	0.8	3.2	2021-07-21	2022-07-06
23884	7mk2	CryoEM Structure of NPR1	0.6	3.8	2021-04-21	2022-03-16
0632	6o6b	Rotavirus A-VP3 (RVA-VP3)	0.024	2.7	2019-03-05	2020-03-11
30999	7e7d	Cryo-EM structure of the SARS-CoV-2 wild-type S-Trimer from a subunit vaccine candidate	0.013	3.2	2021-02-25	2021-03-24
33458	7xu4	Structure of SARS-CoV-2 D614G Spike Protein with Engineered x3 Disulfide (x3(D427C, V987C) and single Arg S1/S2 cleavage site), Locked-2 Conformation	0.022	3.2	2022-05-18	2022-07-20
33456	7xu2	Structure of SARS-CoV-2 Spike Protein with Engineered x3 Disulfide (x3(D427C, V987C) and single Arg S1/S2 cleavage site), Locked-2 Conformation	0.028	3.2	2022-05-18	2022-07-20
22817	7kcq	The Cryo-EM Structure of Alcohol Dehyrogenase from Yeast in Apo Form	0.05	3.2	2020-10-07	2021-03-31
8947	6e0g	Mitochondrial peroxiredoxin from Leishmania infantum after heat stress without unfolding client protein	0.08	2.9	2018-07-06	2019-02-20
27020	8cvx	Human glycogenin-1 and glycogen synthase-1 complex in the presence of glucose-6-phosphate	0.65	3.5	2022-05-18	2022-07-13
6975	5zx5	3.3 angstrom structure of mouse TRPM7 with EDTA	0.013	3.28	2018-05-18	2018-10-17
13752	7q12	Human GYS1-GYG1 complex activated state bound to glucose-6-phosphate	0.0281	3.7	2021-10-17	2022-07-27
31711	7v4h	Cryo-EM Structure of Glycine max glutamine synthetase GmGS Beta2	0.075	2.9	2021-08-13	2022-05-18
7784	6d04	Cryo-EM structure of a Plasmodium vivax invasion complex essential for entry into human reticulocytes; two molecules of parasite ligand, subclass 1.	0.075	3.74	2018-04-10	2018-06-20
30022	6lz3	Structure of cryptochrome in active conformation	0.018	3.2	2020-02-18	2020-04-29

16787	8cpv	Human apoferritin	0.025	1.76	2023-03-03	2023-03-15
0706	6klc	Structure of apo Lassa virus polymerase	0.04	3.9	2019-07-30	2020-03-18
11987	7b2l	Structure of the endocytic adaptor complex AENTH	1.2	3.9	2020-11-27	2021-05-05
25718	7t6l	Cryo-EM structure of TRPV5 at pH5 in nanodiscs	0.136	3.7	2021-12-14	2022-05-04
13053	7osj	ABC Transporter complex NosDFYL, membrane anchor	0.02	3.8	2021-06-08	2022-06-22
10369	6t2u	Cryo-EM structure of the RecBCD in complex with Chi-minus2 substrate	0.02	3.6	2019-10-09	2020-01-01
7850	6dbu	Cryo-EM structure of RAG in complex with 12-RSS and 23-RSS substrate DNAs	0.03	3.9	2018-05-03	2018-08-01
28333	8eob	Cryo-EM structure of human HSP90B in the closed state	3.2	3.1	2022-10-02	2023-01-25
23945	7mqf	Bartonella henselae NrnC complexed with pAAAGG. D4 symmetry.	0.0442	2.88	2021-05-05	2021-09-15
21150	6vd7	Cryo-EM structure of Xenopus tropicalis pannexin 1 channel	3.32	3.02	2019-12-23	2020-02-26
31559	7fep	Cryo-EM structure of BsClpP-ADEP1 complex at pH 6.5	0.8	3.1	2021-07-21	2022-07-06
31561	7fer	Cryo-EM structure of BsClpP-ADEP1 complex at pH 4.2	0.6	3.4	2021-07-21	2022-07-06
7770	6cvm	Atomic resolution cryo-EM structure of beta-galactosidase	0.52	1.9	2018-03-28	2018-05-30
31619	7fje	Cryo-EM structure of a membrane protein(LL)	0.0222	3.0	2021-08-03	2022-07-27
31339	7evp	Cryo-EM structure of the Gp168-beta-clamp complex	0.463	3.2	2021-05-21	2022-02-16
15041	7zzy	Solution BcsD structure	0.105	3.3	2022-05-26	2022-12-28
32382	7wad	Trichodesmium erythraeum cyanophycin synthetase 1 (TeCphA1) with ATPgammaS	0.016	2.96	2021-12-14	2022-09-07
32485	7wgr	Cryo-electron microscopic structure of the 2-oxoglutarate dehydrogenase (E1) component of the human alpha-ketoglutarate (2-oxoglutarate) dehydrogenase complex	0.143	2.92	2021-12-28	2022-06-01
21588	6wbf	Cryo-EM structure of wild type human Pannexin 1 channel	0.012	2.83	2020-03-26	2020-06-03
7785	6d05	Cryo-EM structure of a Plasmodium vivax invasion complex essential for entry into human reticulocytes; two molecules of parasite ligand, subclass 2.	0.073	3.8	2018-04-10	2018-06-20
0560	6nzu	Structure of the human frataxin-bound iron-sulfur cluster assembly complex	0.0425	3.2	2019-02-14	2019-05-22
22757	7ka3	Aldolase, rabbit muscle (beam-tilt refinement x3)	0.0213	3.3	2020-09-29	2020-12-02
30084	6m54	Human apo ferritin frozen on TEM grid with Amorphous nickel titanium alloy supporting film	0.0478	2.4	2020-03-09	2020-05-13
20151	6opo	C3 symmetry reconstruction of CD4- and 17-bound B41 HIV-1 Env SOSIP in complex with DDM	0.5	3.5	2019-04-25	2020-10-21
22805	7kc2	The Cryo-EM Structure of Alcohol Dehyrogenase from Yeast in complex with NADH (open form).	0.032	2.67	2020-10-04	2021-03-31
21577	6w8p	Structure of membrane protein with ions	0.342	3.6	2020-03-21	2021-08-04
13064	7otp	DNA-PKcs in complex with ATPgammaS-Mg	0.23	3.4	2021-06-10	2022-01-12
14149	7qun	CryoEM structure of mammalian AAP in complex with Meropenem	0.5	2.1	2022-01-18	2022-11-16
13052	7osi	ABC Transporter complex NosDFYL, R-domain 3	0.02	3.8	2021-06-08	2022-06-22
26835	7uwl	Structure of the IL-25-IL-17RB-IL-17RA ternary complex	0.036	3.7	2022-05-03	2022-07-27
31105	7eg4	Cryo-EM structure of nauclefine-induced PDE3A-SLFN12 complex	0.0181	3.2	2021-03-24	2021-09-29
23116	7l1r	PS3 F1-ATPase Hydrolysis Dwell	0.15	3.1	2020-12-15	2021-07-21
22048	6x5b	C3 symmetric reconstruction of CD4- and 17-bound B41 HIV-1 Env SOSIP in complex with small molecule GO52	0.02	3.6	2020-05-25	2020-10-21
28273	8enm	CryoEM structure of the high pH nitrogenase MoFe-protein under non-turnover conditions	0.315	2.14	2022-09-30	2023-03-08
24987	7sbs	One RBD-up 1 of pre-fusion SARS-CoV-2 Gamma variant spike protein	0.1	3.8	2021-09-25	2021-11-03
23948	7mqi	Bartonella henselae NrnC complexed with pAAAGG in the presence of Ca2+. C1 reconstruction.	0.0762	3.21	2021-05-05	2021-09-15
0740	6kou	277 K cryoEM structure of Sso-KARI in complex with magnesium ions	1.48	2.43	2019-08-13	2020-03-25

20212	6ovh	Cryo-EM structure of Bimetallic dodecameric cage design 3 (BMC3) from cytochrome cb562	0.026	2.6	2019-05-07	2020-01-29
11185	6zfp	Cryo-EM structure of DNA-PKcs (State 2)	0.055	3.24	2020-06-17	2020-10-21
24982	7sbl	One RBD-up 1 of pre-fusion SARS-CoV-2 Delta variant spike protein	0.1	3.4	2021-09-25	2021-11-10
33459	7xu5	Structure of SARS-CoV-2 D614G Spike Protein with Engineered x3 Disulfide (x3(D427C, V987C) and single Arg S1/S2 cleavage site), Closed Conformation	0.022	3.1	2022-05-18	2022-07-20
22221	6xkl	SARS-CoV-2 HexaPro S One RBD up	0.45	3.21	2020-06-26	2020-07-15
11210	6zgl	Structure of DPS determined by movement-free cryoEM with zero dose extrapolation	0.015	1.9	2020-06-19	2020-10-21
30707	7dkj	Hemagglutinin Influenza A virus (A/Okuda/1957(H2N2) bound with a neutralizing antibody	0.06	3.7	2020-11-24	2021-11-24
9039	6ee8	Mycobacterium tuberculosis RNAP promoter unwinding intermediate complex with RbpA/CarD and AP3 promoter	0.3	3.92	2018-08-13	2018-11-21
13049	7osf	ABC Transporter complex NosDFYL, R-domain 1	0.022	3.8	2021-06-08	2022-06-22
24238	7n8n	Melbournevirus nucleosome like particle	0.116	3.89	2021-06-15	2021-08-04
21589	6wbg	Cryo-EM structure of human Pannexin 1 channel with its C-terminal tail cleaved by caspase-7	0.016	2.97	2020-03-26	2020-06-03
14705	7zg7	Structure of human Apoferritin obtained from ssDNA coated grid	0.02	1.77	2022-04-02	2022-11-23
32381	7wac	Trichodesmium erythraeum cyanophycin synthetase 1 (TeCphA1)	0.015	2.91	2021-12-14	2022-09-07
30811	7dpw	Structural basis for ligand binding modes of CTP synthase	0.05	2.65	2020-12-21	2021-09-15
15960	8bc2	Ligand-Free Structure of the decameric sulfofructose transaldolase BmSF-TAL	0.0304	2.6	2022-10-14	2023-01-18
11776	7ag8	Cryo-EM structure of wild-type KatG from M. tuberculosis	0.596	2.68	2020-09-21	2021-01-27
31432	7f3e	Cryo-EM structure of the minimal protein-only RNase P from Aquifex aeolicus reveals structural insight into precursor tRNA recognition and catalysis	0.06	3.62	2021-06-16	2021-08-11
27070	8cy8	apo form Cryo-EM structure of Campylobacter jejune ketol-acid reductoisommerase crosslinked by Glutaraldehyde	0.41	2.94	2022-05-23	2023-02-01
13051	7osh	ABC Transporter complex NosDFYL, R-domain 2	0.02	3.8	2021-06-08	2022-06-22
30021	6lz1	Structure of S.pombe alpha-mannosidase Ams1	0.03	3.2	2020-02-17	2020-09-09
31103	7eg0	Cryo-EM structure of anagrelide-induced PDE3A-SLFN12 complex	0.02	3.4	2021-03-23	2021-09-29
10347	6syt	Structure of the SMG1-SMG8-SMG9 complex	0.07	3.45	2019-10-01	2019-12-11
24123	7n1u	Structural basis for enhanced infectivity and immune evasion of SARS-CoV-2 variants	0.25	3.14	2021-05-28	2021-07-07
14147	7quc	D. melanogaster alpha/beta tubulin heterodimer in the GDP form	0.0475	3.2	2022-01-17	2022-09-21
26477	7ufm	VchTnsC AAA+ with DNA (double heptamer)	0.15	3.9	2022-03-22	2022-06-08
22756	7ka2	Aldolase, rabbit muscle (beam-tilt refinement x2)	0.0212	3.6	2020-09-29	2020-12-02
22278	6xos	CryoEM structure of human presequence protease in partial open state 1	0.005	3.7	2020-07-07	2021-07-07
23594	7lyl	South African (B.1.351) SARS-CoV-2 spike protein variant (S-GSAS-B.1.351) in the RBD-down conformation	0.2	3.72	2021-03-07	2021-03-31
0683	6j5w	Ligand-triggered allosteric ADP release primes a plant NLR complex	0.03	3.7	2019-01-12	2019-04-03
13743	7q0b	Human GYS1-GYG1 complex inhibited state	0.021	3.0	2021-10-14	2022-07-27
11331	6zoz	Structure of Disulphide-stabilized SARS-CoV-2 Spike Protein Trimer (x1 disulphide-bond mutant, S383C, D985C, K986P, V987P, single Arg S1/S2 cleavage site) in Locked State	0.0118	3.5	2020-07-08	2020-07-22
10279	6sp2	CryoEM structure of SERINC from Drosophila melanogaster	0.7	3.33	2019-08-30	2020-01-01
13075	7ouf	Structure of the STLV intasome:B56 complex bound to the strand-transfer inhibitor XZ450	0.0177	3.0	2021-06-11	2021-08-18

29028	8fei	CryoEM structure of Conalbumin from chicken egg white (sigma-Cas 1391-06-6)	0.278	3.0	2022-12-06	2023-02-08
10402	6t8o	Stalled FtsK motor domain bound to dsDNA end	0.0205	3.99	2019-10-24	2019-11-20
31469	7f5x	GK domain of Drosophila P5CS filament with glutamate	0.01	3.5	2021-06-23	2022-04-06
13035	7oqz	Cryo-EM structure of human TMEM45A	0.2	3.27	2021-06-04	2021-06-16
11215	6zh6	Cryo-EM structure of DNA-PKcs:Ku80ct194	0.125	3.93	2020-06-21	2020-10-21
14522	7z5j	The molybdenum storage protein loaded with tungstate	0.0082	2.58	2022-03-09	2022-07-13
26957	8crs	CryoEM Structure of nitrogenase MoFe-protein in detergent	0.29	2.04	2022-05-11	2023-03-08
11213	6zh4	Cryo-EM structure of DNA-PKcs (State 3)	0.055	3.62	2020-06-20	2020-10-21
30995	7e5z	Dehydrogenase holoenzyme	0.164	3.6	2021-02-21	2022-02-23
10216	6sjf	Cryo-EM structure of the RecBCD Chi unrecognised complex	0.011	3.9	2019-08-13	2020-01-01
0990	6lvd	Structure of Dimethylformamidase, tetramer, Y440A mutant	0.0638	3.2	2020-02-02	2020-06-03
31491	7f8o	Cryo-EM structure of the C-terminal deletion mutant of human PANX1 in a nanodisc	0.01	3.6	2021-07-02	2022-01-26
24122	7n1t	Structural basis for enhanced infectivity and immune evasion of SARS-CoV-2 variants	0.25	3.11	2021-05-28	2021-07-07
24347	7ra8	SARS-CoV-2 S glycoprotein in complex with S2X259 Fab	0.35	3.1	2021-06-30	2021-08-04

Supplementary Table S7: Description of test dataset used from Cryo2StructData

EMD ID	PDB ID	Title	Contour Level	Resolution	Deposition Date	Release Date
15691	8aw5	Cryo-EM structure of heme A synthase trimer from Aquifex aeolicus	0.0488	2.8	2022-08-29	2023-09-06
16110	8blo	Human Urea Transporter UT-A (N-Terminal Domain Model)	0.7	2.9	2022-11-10	2023-10-04
16112	8blp	Human Urea Transporter UT-B/UT1 in Complex with Inhibitor UTBinh-14	0.9	2.6	2022-11-10	2023-10-04
16140	8bob	Structural basis for negative regulation of the maltose system	0.015	2.94	2022-11-15	2023-10-18
16328	8bym	Outer membrane attachment porin OmpM1 from Veillonella parvula	0.1	3.15	2022-12-13	2023-11-08
16332	8bys	Outer membrane attachment porin OmpM1 from Veillonella parvula, native	0.241	3.28	2022-12-13	2023-11-08
16333	8byt	Outer membrane attachment porin OmpM1 from Veillonella parvula, C3 symmetry	0.179	2.78	2022-12-13	2023-11-08
16475	8c8g	Cryo-EM structure of BoNT/Wo-NTNH complex	0.124	2.98	2023-01-20	2023-10-04
17360	8p2c	Cryo-EM structure of the anaerobic ribonucleotide reductase from Prevotella copri in its tetrameric state produced in the presence of dATP and CTP	0.165	2.59	2023-05-15	2023-09-13
17402	8p49	Uncharacterized Q8U0N8 protein from Pyrococcus furiosus	0.05	2.79	2023-05-19	2023-11-29
17429	8p4x	FAD_ox bound dark state structure of PdLCry	1.75	2.57	2023-05-23	2023-11-08
17574	8p97	BtuB3G3 bound to cyanocobalamin with disordered EL8	0.163	2.75	2023-06-05	2023-08-16
17575	8p98	BtuB3G3 bound to cyanocobalamin with ordered EL8	0.113	2.97	2023-06-05	2023-08-16
17958	8pv9	Structure of DPS determined by cryoEM at 100 keV	0.04	2.7	2023-07-17	2023-11-29
17961	8pvc	Structure of mouse heavy-chain apoferritin determined by cryoEM at 100 keV	0.04	2.6	2023-07-17	2023-11-29
17964	8pvf	Structure of GAPDH determined by cryoEM at 100 keV	0.04	2.9	2023-07-17	2023-11-29
17966	8pvh	Structure of human apo ALDH1A1 determined by cryoEM at 100 keV	0.035	2.9	2023-07-17	2023-11-29
18148	8q4h	a membrane-bound menaquinol:organohalide oxidoreductase complex RDH complex	0.102	2.83	2023-08-07	2023-10-18
18212	8q7c	Cryo-EM structure of Adenovirus C5 hexon	0.236	2.9	2023-08-16	2023-08-30
18298	8qa4	MTHFR + SAH symmetric dis-inhibited state	0.07	2.8	2023-08-22	2023-11-08
18300	8qa6	MTHFR + SAM inhibited state	0.2	2.91	2023-08-22	2023-11-08
18415	8qhp	Cysteine tRNA ligase homodimer	0.035	2.8	2023-09-08	2023-11-29

18436	8qi7	Cryo-EM Structure of Human Serine Hydroxymethyltransferase, isoform 2 (SHMT2)	0.19	2.9	2023-09-11	2023-09-20
18634	8qsk	Cryo-EM structure of human SLC15A4 dimer in outward open state in MSP1D1 nanodisc	0.18	3.3	2023-10-10	2023-11-01
18635	8qsl	Cryo-EM structure of human SLC15A4 dimer in outward open state in LMNG	0.207	2.81	2023-10-10	2023-10-25
26165	7ty4	Cryo-EM structure of human Anion Exchanger 1	0.4	2.99	2022-02-11	2023-08-16
28628	8evg	162bp CX3CR1 nucleosome (further classified with better nucleosome end)	0.5	2.75	2022-10-20	2023-11-01
28994	8fcv	Cryo-EM structure of TnsC-TniQ-DNA complex in type I-B CAST system	2.23	2.95	2022-12-01	2023-08-09
29069	8ffw	Cryo-EM structure of the GR-Hsp90-FKBP51 complex	0.15	3.23	2022-12-10	2023-11-01
29551	8fy3	Structure of NOT1:NOT10:NOT11 module of the human CCR4-NOT complex	0.522	2.88	2023-01-25	2023-07-26
29552	8fy4	Structure of NOT1:NOT10:NOT11 module of the chicken CCR4-NOT complex	0.232	2.57	2023-01-25	2023-08-30
29823	8g7t	Cryo-EM structure of RNP end	0.008	3.2	2023-02-17	2023-11-15
29930	8gcc	T. cruzi topoisomerase II alpha bound to dsDNA and the covalent inhibitor CT1	0.6	2.94	2023-03-01	2023-07-12
33592	7y3e	Cryo-EM structure of Arabidopsis thaliana SOS1 in an occluded state	0.5	2.8	2022-06-10	2023-08-09
33639	7y6f	Cryo-EM structure of Apo form of ScBfr	0.027	2.7	2022-06-20	2023-07-05
34219	8gs3	Cryo-EM structure of human Neuroligin 3	0.01	3.9	2022-09-04	2023-09-20
34275	8guk	Human nucleosome core particle (free form)	0.4	2.51	2022-09-12	2023-09-20
34304	8gw7	AtSLAC1 6D mutant in open state	0.139	3.3	2022-09-16	2023-11-15
34368	8gy2	Cryo-EM Structure of Membrane-Bound Alcohol Dehydrogenase from Gluconobacter oxydans	0.16	2.5	2022-09-21	2023-08-02
34369	8gy3	Cryo-EM Structure of Membrane-Bound Aldehyde Dehydrogenase from Gluconobacter oxydans	0.12	2.7	2022-09-21	2023-08-02
34412	8h0i	Cryo-EM structure of APOBEC3G-Vif complex	4	2.8	2022-09-29	2023-07-19
34504	8h6h	cryo-EM structure of cellodextrin phosphorylase from Clostridium thermocellum	0.3	2.3	2022-10-17	2023-10-25
34825	8hip	dsRNA transporter	0.962	2.77	2022-11-21	2023-11-29
35075	8hxb	Cryo-EM structure of MPXV M2 hexamer in complex with human B7.2	0.15	2.7	2023-01-04	2023-08-30
35452	8ihq	Cryo-EM structure of ochratoxin A-detoxifying amidohydrolase ADH3	0.25	2.71	2023-02-23	2023-08-30
35453	8ihr	Cryo-EM structure of ochratoxin A-detoxifying amidohydrolase ADH3 in complex with Phe	0.25	2.5	2023-02-23	2023-08-30
35454	8ihs	Cryo-EM structure of ochratoxin A-detoxifying amidohydrolase ADH3 in complex with ochratoxin A	0.25	2.5	2023-02-23	2023-08-30
35621	8iol	The complex of Rubisco large subunit (RbcL)	0.034	2.9	2023-03-11	2023-11-01
35713	8iu0	Cryo-EM structure of the potassium-selective channelrhodopsin HcKCR1 H225F mutant in lipid nanodisc	0.613	2.66	2023-03-23	2023-09-06
36060	8j85	Cryo-EM structure of ochratoxin A-detoxifying amidohydrolase ADH3 mutant S88E in complex with ochratoxin A	0.25	2.7	2023-04-30	2023-08-30
36150	8jc7	Cryo-EM structure of Vibrio campbellii alpha-hemolysin	0.5	2.06	2023-05-10	2023-09-27
36391	8jlb	Cryo-EM structure of the 145 bp human nucleosome containing H3.2 C110A mutant	0.06	2.36	2023-06-02	2023-10-04
36628	8jsw	Human VMAT2 complex with serotonin	1.26	2.84	2023-06-20	2023-11-29
36637	8jt9	Human VMAT2 complex with ketanserin	0.383	2.97	2023-06-21	2023-11-29
36661	8jul	Cryo-EM structure of SIDT1 in complex with phosphatidic acid	0.474	2.92	2023-06-26	2023-11-15
36662	8jun	Cryo-EM structure of SIDT1 E555Q mutant	0.475	2.38	2023-06-26	2023-11-15
36856	8k3q	S. cerevisiae Chs1 in apo state	0.3	2.6	2023-07-16	2023-10-18

36863	8k3w	S. cerevisiae Chs1 in complex with UDP-GlcNAc and GlcNAc	0.19	2.91	2023-07-17	2023-10-18
36864	8k3x	S. cerevisiae Chs1 in complex with Nikkomycin Z	0.623	2.86	2023-07-17	2023-10-18
37235	8kgy	Human glutamate dehydrogenase I	0.294	2.59	2023-08-20	2023-10-25
40039	8ghb	The structure of h12-LOX in monomeric form	0.45	2.76	2023-03-09	2023-08-09
40040	8ghc	The structure of h12-LOX in dimeric form	11	2.3	2023-03-09	2023-08-09
40041	8ghd	The structure of h12-LOX in hexameric form bound to inhibitor ML355 and arachidonic acid	11	2.2	2023-03-09	2023-08-09
40063	8gi9	Cation channelrhodopsin from Hyphochytrium catenoides (HcCCR) embedded in peptidisc	0.6	2.84	2023-03-13	2023-07-26
40229	8gmp	Cryo-EM structure of octameric human CALHM1 with a I109W point mutation	0.154	2.8	2023-03-27	2023-07-26
40334	8sc1	Human OCT1 (Apo) in inward-open conformation	0.235	2.92	2023-04-04	2023-10-18
40335	8sc2	Human OCT1 bound to diltiazem in inward-open conformation	0.123	3.36	2023-04-04	2023-10-18
40336	8sc3	Human OCT1 bound to fenoterol in inward-open conformation	0.1	3.24	2023-04-04	2023-10-18
40339	8sc6	Human OCT1 bound to thiamine in inward-open conformation	0.238	3.13	2023-04-04	2023-10-18
40352	8sdu	Structure of rat organic anion transporter 1 (OAT1)	0.3	2.05	2023-04-07	2023-10-18
40354	8sdy	Structure of rat organic anion transporter 1 (OAT1) in complex with para-aminohippuric acid (PAH)	0.01	2.79	2023-04-07	2023-10-18
40355	8sdz	Structure of rat organic anion transporter 1 (OAT1) in complex with probenecid	0.01	2.86	2023-04-07	2023-10-18
40889	8syp	Genomic CX3CR1 nucleosome	0.16	2.6	2023-05-25	2023-11-01
41066	8t69	Human VMAT2 in complex with tetrabenazine	0.386	2.89	2023-06-15	2023-11-01
41266	8thj	Cryo-EM structure of the Tripartite ATP-independent Periplasmic (TRAP) transporter SiaQM from Haemophilus influenzae (antiparallel dimer)	0.05	2.99	2023-07-16	2023-11-22
41604	8ttb	Cryo-EM structure of the PP2A:B55-ARPP19 complex	0.0075	2.77	2023-08-13	2023-10-25
41624	8tul	Cryo-EM structure of the human MRS2 magnesium channel under Mg2+ condition	1.6	2.8	2023-08-16	2023-09-13
41628	8tup	Cryo-EM structure of the human MRS2 magnesium channel under Mg2+-free condition	0.14	3.3	2023-08-16	2023-09-13
41768	8tzs	Structure of human WLS	0.117	3.84	2023-08-27	2023-10-18
41946	8u66	Firmicutes Rubisco	0.425	2.21	2023-09-13	2023-11-22

Supplementary Table S8: Description of standard test dataset

EMD ID	PDB ID	Title	Contour Level	Resolution	Deposition Date	Release Date
14066	7qla	Structure of the Rab GEF complex Mon1-Ccz1	0.02	3.85	2021-12-20	2022-02-09
14716	7zh0	Structure of human OCT3 in lipid nanodisc	0.0113	3.2	2022-04-05	2022-11-09
14725	7zh6	Structure of human OCT3 in complex with inhibitor Corticosterone	0.008	3.67	2022-04-05	2022-11-09
14842	7zny	Cryo-EM structure of the canine distemper virus tetrameric attachment glycoprotein	0.65	3.26	2022-04-23	2023-02-08
14847	7zol	Cryo-EM structure of a CRISPR effector in complex with regulator	0.15	3.03	2022-04-26	2022-11-30
14848	7zoq	Cryo-EM structure of a CRISPR effector in complex with a caspase regulator	0.1	3.2	2022-04-26	2022-11-30
14869	7zqb	Tail tip of siphophage T5 : full structure	0.028	3.88	2022-04-29	2023-02-08
15378	8ae1	Structure of trimeric SlpA outer membrane protein	0.025	3.25	2022-07-12	2022-11-30
15540	8ane	Structure of the type I-G CRISPR effector	0.0103	3.2	2022-08-05	2022-11-09
15635	8at6	Cryo-EM structure of yeast Elp456 subcomplex	0.0242	3.7	2022-08-22	2022-12-07
15673	8aur	Cryo-EM structure of a TasA fibre	0.022	3.47	2022-08-25	2022-11-30

15684	8avv	Cryo-EM structure of DrBphP photosensory module in Pr state	0.09	3.4	2022-08-27	2022-12-21
15685	8avw	Cryo-EM structure of DrBphP in Pr state	0.095	3.62	2022-08-27	2022-12-21
15686	8avx	Cryo-EM structure of DrBphP in Pfr state	0.13	3.5	2022-08-27	2022-12-21
15785	8b0j	CryoEM structure of bacterial RNaseE.RapZ.GlmZ complex central to the control of cell envelope biogenesis	0.04	3.99	2022-09-07	2022-10-05
23544	7lw1	Human phosphofructokinase-1 liver type bound to activator NA-11	1.1	2.9	2021-02-27	2022-01-26
26595	7ulw	CryoEM structure of human LACTB filament	0.0042	3.1	2022-04-05	2022-12-07
26754	7ut4	Gea2 closed/closed conformation (composite structure)	5.85	3.9	2022-04-26	2022-08-24
26770	7uth	Gea2 open/open conformation (composite structure)	6	3.9	2022-04-26	2022-08-24
26782	7utn	IscB and wRNA bound to Target DNA	0.135	2.74	2022-04-27	2022-06-15
26841	7uws	Atomic model of the partial VSV nucleocapsid	0.025	3.47	2022-05-03	2022-09-28
26858	7uxe	Pseudomonas phage E217 small terminase (TerS)	0.5	3.38	2022-05-05	2022-09-28
26917	7uzs	Protein 4.2 (local refinement from consensus reconstruction of ankyrin complex classes)	1	2.2	2022-05-09	2022-07-20
26948	7v0q	Local refinement of protein 4.2, class 1 of erythrocyte ankyrin-1 complex	1	2.5	2022-05-10	2022-07-20
26973	8csw	Local refinement of protein 4.2 in Class 2 of erythrocyte ankyrin-1 complex	1	2.5	2022-05-13	2022-07-20
26974	8csx	Local refinement of RhAG/CE trimer in class 2 of erythrocyte ankyrin-1 complex	1	2.4	2022-05-13	2022-07-20
26976	8csz	IscB and wRNA bound to Target DNA	0.158	3.2	2022-05-13	2022-06-15
26978	8ct2	Local refinement of AQP1 tetramer (C1; refinement mask included D1 of protein 4.2 and Ankyrin-1 AR1-5) in Class 2 of erythrocyte ankyrin-1 complex	0.6	3.1	2022-05-13	2022-07-20
26993	8ctk	Cryo-EM structure of SARS-CoV-2 M protein in a lipid nanodisc	0.17	3.52	2022-05-15	2022-06-22
26994	8ctl	IscB and wRNA bound to Target DNA (locked state)	0.0924	3.1	2022-05-16	2022-06-15
27138	8d1v	Cryo-EM structure of guide RNA and target RNA bound Cas7-11	0.0146	2.82	2022-05-27	2022-11-02
27252	8d8n	gRAMP non-match PFS target RNA	0.196	3.6	2022-06-08	2022-08-31
27253	8d8o	Structure of a metalloprotease.	0.2298	3.35	2022-06-08	2022-09-28
27320	8dc2	Cryo-EM structure of CasLambda (Cas12l) bound to crRNA and DNA	10	2.99	2022-06-15	2022-12-14
27574	8dnm	Human Brain Dihydropyrimidinase-related protein 2	0.2	2.76	2022-07-11	2022-11-16
27645	8dq0	Quorum-sensing receptor RhlR bound to PqsE	0.35	3.74	2022-07-18	2022-12-07
27656	8dql	CryoEM structure of IglD	0.0345	3.0	2022-07-19	2022-08-17
27661	8dqv	The 1.52 angstrom CryoEM structure of the [NiFe]-hydrogenase Huc from Mycobacterium smegmatis - catalytic dimer (Huc2S2L)	0.612	1.52	2022-07-20	2023-01-04
27755	8dwi	Molecular Mechanism of Sialic Acid Transport Mediated by Sialin	0.155	3.4	2022-08-01	2023-01-25
27758	8dws	Full-length E47K SPOP	0.18	3.73	2022-08-02	2023-01-18
27760	8dwu	SPOP W22R Hexameric form	0.5	3.4	2022-08-02	2023-01-18
27761	8dwv	Full-length wild type SPOP	0.5	3.6	2022-08-02	2023-01-18
27899	8e50	Cryo-EM structure of human glycerol-3-phosphate acyltransferase 1 (GPAT1) in complex with CoA and palmitoyl-LPA	0.015	3.67	2022-08-19	2022-12-21
28064	8eex	Cas7-11 in complex with Csx29, focus refined on Cas7-11	0.02	2.95	2022-09-07	2022-11-16
28065	8eey	Cas7-11 in complex with DR-mismatched target RNA, Csx29 and Csx30	0.02	2.53	2022-09-07	2022-11-16
28641	8ew3	Cryo EM structure of Vibrio cholerae NQR	0.016	2.65159	2022-10-21	2022-11-16
28660	8exr	Cryo-EM structure of S. aureus BlaR1 TM and zinc metalloprotease domain	0.07	3.8	2022-10-25	2023-01-11

28666	8ey2	Cryo-EM structure of SARS-CoV-2 Main protease C145S in complex with N-terminal peptide	6.44	3.5	2022-10-26	2022-12-07
28866	8f5o	Structure of Leishmania tarentolae IFT-A (state 1)	9	3.5	2022-11-14	2022-12-21
28867	8f5p	Structure of Leishmania tarentolae IFT-A (state 2)	7	3.4	2022-11-14	2022-12-21
33187	7xgr	Structure of Gemin5 C-terminal region (protomer)	0.14	2.6	2022-04-06	2022-08-24
33233	7xjp	Cryo-EM structure of EDS1 and SAG101 with ATP-APDR	0.01	2.71	2022-04-18	2022-07-20
33242	7xk3	Cryo-EM structure of Na+-pumping NADH-ubiquinone oxidoreductase from Vibrio cholerae, state 1	0.5	3.1	2022-04-19	2022-07-20
33243	7xk4	Cryo-EM structure of Na+-pumping NADH-ubiquinone oxidoreductase from Vibrio cholerae, state 2	0.55	3.1	2022-04-19	2022-07-20
33244	7xk5	Cryo-EM structure of Na+-pumping NADH-ubiquinone oxidoreductase from Vibrio cholerae, state 3	0.5	3.1	2022-04-19	2022-07-20
33245	7xk6	Cryo-EM structure of Na+-pumping NADH-ubiquinone oxidoreductase from Vibrio cholerae, with aurachin D-42	0.7	3.0	2022-04-19	2022-07-20
33331	7xnz	Cyo-EM model for native cystathionine beta-synthase of Mycobacterium tuberculosis.	0.0292	3.6	2022-04-30	2022-05-25
33348	7xoh	Cryo-EM map of cystathionine beta-synthase of Mycobacterium tuberculosis in the presence of S-adenosylmethionine.	0.0348	3.6	2022-05-01	2022-05-25
33430	7xsp	Structure of gRAMP-target RNA	0.025	2.89	2022-05-15	2022-11-09
33431	7xsq	Structure of the Craspase	0.027	2.88	2022-05-15	2022-11-09
33432	7xsr	Structure of Craspase-target RNA	0.036	2.97	2022-05-15	2022-11-09
33433	7xss	Structure of Craspase-CTR	0.022	3.2	2022-05-15	2022-11-09
33439	7xt4	Structure of Craspase-NTR	0.056	3.08	2022-05-16	2022-11-09
33528	7xzi	Cryo-EM structure of TOC-TIC supercomplex from Chlamydomonas reinhardtii	0.15	2.77	2022-06-02	2023-01-11
33676	7y80	CryoEM structure of type III-E CRISPR gRAMP-crRNA binary complex	1.2	2.71	2022-06-22	2022-12-14
33677	7y81	CryoEM structure of type III-E CRISPR gRAMP-crRNA complex bound to non-self RNA target	1.3	2.54	2022-06-22	2022-12-14
33678	7y82	CryoEM structure of type III-E CRISPR gRAMP-crRNA complex bound to self RNA target	1	2.83	2022-06-22	2022-12-14
33853	7yi8	Cryo-EM structure of SAH-bound MTA1-MTA9-p1-p2 complex	0.3	2.7	2022-07-15	2023-01-18
33854	7yi9	Cryo-EM structure of SAM-bound MTA1-MTA9-p1-p2 complex	0.35	2.6	2022-07-15	2023-01-18
33955	7yn9	Cryo-EM structure of Cas7-11-crRNA binary complex	0.012	3.53	2022-07-30	2023-02-01
33956	7yna	Cryo-EM structure of Cas7-11-crRNA bound to target RNA-1	0.02	3.64	2022-07-30	2023-02-01
33957	7ynb	Cryo-EM structure of Cas7-11-crRNA bound to target RNA-2	0.018	3.46	2022-07-30	2023-02-01
33958	7ync	Cryo-EM structure of Cas7-11-crRNA bound to target RNA-3	0.0157	3.14	2022-07-30	2023-02-01
33959	7ynd	Cryo-EM structure of Cas7-11-crRNA-Csx29 ternary complex	0.0192	3.29	2022-07-30	2023-02-01
34017	7ypx	Cyanophage Pam3 fiber	0.2	3.12	2022-08-04	2022-11-09
34023	7yqc	EM structure of human PA28gamma	0.4	2.82	2022-08-06	2022-09-21
34024	7yqd	EM structure of human PA28gamma (wild-type)	0.2	3.4	2022-08-06	2022-09-21
34158	8gna	Structure of the SbCas7-11-crRNA-NTR complex	6.5	2.8	2022-08-23	2023-01-18
34270	8gu6	Structure of the SbCas7-11-crRNA-NTR-Csx29 complex	0.2	3.1	2022-09-10	2023-01-18
34738	8hgg	Structure of 2:2 PAPP-A.ProMBP comple	0.0262	3.64	2022-11-14	2023-01-11
